# A Re-Description of *‘Mycterosaurus’ smithae*, an Early Permian Eothyridid, and Its Impact on the Phylogeny of Pelycosaurian-Grade Synapsids

**DOI:** 10.1371/journal.pone.0156810

**Published:** 2016-06-22

**Authors:** Neil Brocklehurst, Robert R. Reisz, Vincent Fernandez, Jörg Fröbisch

**Affiliations:** 1 Museum für Naturkunde, Leibniz-Institut für Evolutions- und Biodiversitätsforschung, Invalidenstraße 43, D-10115, Berlin, Germany; 2 Department of Biology, University of Toronto Mississauga, 3359 Mississauga Rd., Mississauga, L5L 1C6, Canada; 3 European Synchrotron Radiation Facility, 71 Avenue des Martyrs, 38000, Grenoble, France; 4 Institut für Biologie, Humboldt-Universität zu Berlin, Invalidenstraße 110, Berlin, D-10115, Germany; New York Institute of Technology College of Osteopathic Medicine, UNITED STATES

## Abstract

*‘Mycterosaurus’ smithae*, from the Cisuralian (early Permian) of Colorado, was first described in 1965 as a second species of the genus *Mycterosaurus*. While the type species of this genus, *M*. *longiceps*, has been shown by multiple cladistic analyses to belong to the basal synapsid family Varanopidae, *‘M*.*’ smithae* has been largely ignored since its original description. Additional preparation and synchrotron scanning has revealed new significant information that supports the assignment of this species to a new genus: *Vaughnictis* gen. nov. *Vaughnictis* lacks many of the characteristics of mycterosaurines and varanopids in general: it lacks the slender femur, the linguo-labially compressed and strongly recurved teeth, and the lateral boss on the postorbital characteristic of this family. Instead, it possesses coronoid teeth, a large supratemporal, and a large pineal foramen positioned midway along the length of the parietal, features that support its assignment to Eothyrididae. Moreover, the postcranium shares many characters with the eothyridid *Oedaleops*. An expanded version of a recently published phylogenetic analysis of pelycosaurian-grade synapsids positions *Vaughnictis* as the sister taxon of *Eothyris* within the clade Eothyrididae. The addition of data on the postcranium of eothyridids and the inclusion of the recently-described basal caseid *Eocasea* confirms the recently-disputed position of caseasaurs as the most basal synapsids. As the parsimony analysis produced low support values and a lack of resolution due to missing data, additional analyses were undertaken using Bayesian and Implied Weights methods, which produced better resolution and relationships with higher support values. While the results are similar, alternative positions for the enigmatic Moscovian age (Carboniferous) synapsid *Echinerpeton* are suggested by Bayesian analysis; the parsimony analysis found it to be an ophiacodontid, while the Bayesian and Implied Weights analysis found it to be the sister to the Sphenacomorpha.

## Introduction

Eothyrididae is a clade of Cisuralian (early Permian) synapsids, belonging to the paraphyletic grade of six families known as the Pelycosauria. For many years after this family was erected [1], it was treated mainly as a ‘wastebasket taxon’, filled with any small, carnivorous pelycosaurian-grade synapsid that did not seem to fit into any of the other families. Nine genera were included within Eothyrididae in the review of Langston [2], but with the introduction of a classification based on cladistics most were assigned to other families either within Synapsida or in some cases more distant clades [3]. Only two species were retained: *Eothyris parkeyi* [4] from the Artinskian-lower Kungurian Belle Plains Formation of Texas, and *Oedaleops campi* [2] from the upper El Cobre Canyon Formation of New Mexico, probably of Asselian or early Sakmarian age. The monophyly of the family containing these two species has been confirmed by phylogenetic analyses [5,6], although Sumida et al. [7] suggested the eothyridids formed a paraphyletic grouping within Caseasauria.

In 1965, Lewis and Vaughn published a description of the vertebrate fauna found at the Placerville localities of south-western Colorado [8]. Among the material were two specimens assigned to a new species of *Mycterosaurus*: *M*. *smithae*. The holotype (MCZ 2985) consisted of a partial skull, five vertebrae and ribs and a proximal femur and tibia, while the referred specimen (USNM 22098) was a partial femur and a string of seven vertebrae. The type species of *Mycterosaurus*, *M*. *longiceps* [9], has been included in numerous cladistic analyses which have supported its assignment to the varanopid subfamily Mycterosaurinae [6,10–13]. However *M*. *smithae* has received comparatively little attention since its original description.

The re-examination of the holotype of *M*. *smithae* presented here supports its affinity with Eothyrididae. Additional preparation has revealed considerably more detail of both the skull and the postcranium, the latter representing only the second example of postcranial material from an eothyridid. The specimen was included in a modified and expanded version of a previously published matrix examining the global relationships of pelycosaurian-grade synapsids [6], which was analysed using multiple methods in order to compare the results and resolve certain debates surrounding the phylogeny of pelycosaurs. The phylogenies produced from this matrix were analysed in order to determine the causes of instability within the trees.

## Materials and Methods

### Propagation Phase Contrast Synchrotron Radiation micro-Computed Tomography

The skull of MCZ 2985 was analysed using Propagation Phase Contrast Synchrotron Radiation micro-Computed Tomography (PPC-SR-μCT) at the BM05 beamline of the European Synchrotron Radiation Facility (ESRF, Grenoble France) using filtered white beam (Al: 3 mm, Cu: 3 mm) with a total integrated energy of about 105 keV. The sample-detector distance was about 4.2 m to observe sufficient phase contrast effect by free-air propagation. The optic setup consisted of a100 μm GGG scintilaltor, a 0.5X magnification set of lenses and a PCO.edge camera, resulting in a measured 12.02 μm isotropic pixel size on the recorded radiographs. 8000 projections were recorded over 360 degrees in order to compensate for the noise of the camera, with an exposure time of 30 ms each (as well as 201 flat field images and 200 dark field images). The centre of rotation was set 500 pixels to the right of the images to increase the final horizontal field of view in the final tomograms (so called half-acquisition protocol). Given vertical size of the incident beam (~3.6 mm), 36 scans were needed to cover the full length of the skull, with a vertical displacement of 1.8 mm between two consecutive scans (50% of overlap to compensate for the vertical intensity profile of the X-ray beam). Tomographic reconstruction was performed using filtered-back projection from the PyHST2 software with Paganin approach for single distance phase retrieval [14,15]. The generated stacks of 32bits EDF files were converted to 16 bit stacks of TIF (using min and max 32 bits crop values from the 3D histogram provided by PyHST2). For the final vertical concatenations of the series of scans, we took advantage of the important overlap between consecutive scans and performed a weighted average of similar slices, giving more weight to a slice when it was closer to the centre of the stack than to the border (as the intensity of the X-ray beam is greater at the centre).

### Nomenclatural acts

The electronic edition of this article conforms to the requirements of the amended International Code of Zoological Nomenclature, and hence the new names contained herein are available under that Code from the electronic edition of this article. This published work and the nomenclatural acts it contains have been registered in ZooBank, the online registration system for the ICZN. The ZooBank LSIDs (Life Science Identifiers) can be resolved and the associated information viewed through any standard web browser by appending the LSID to the prefix “http://zoobank.org/”. The LSID for this publication is: urn:lsid:zoobank.org:pub:89CDF14F-0EBB-4C7B-B586-D25532C68238. The electronic edition of this work was published in a journal with an ISSN, and has been archived and is available from the following digital repositories: PubMed Central, LOCKSS.

### Geological Setting

The Cutler Group spans the late Pennsylvanian (Carboniferous) and much of the Early Permian [16], outcropping across New Mexico, Utah and Colorado. The Placerville area, from which MCZ 2985 originates, is a locality in Colorado where the strata of the Cutler Group are exposed in the San Miguel Canyon [8]. Unfortunately, the biostratigraphy of the Cutler Group in Colorado is not as well established as in other areas. Lewis & Vaughn [8] considered the localities to represent the upper portion of the Cutler Group, equivalent to the late Sakmarian-Artinskian aged Moran, Putnam and Admiral Formations [16]. However, they also drew comparisons with the Dunkard Group of Ohio. Most of the taxa from Placerville which are shared with the Dunkard Group are found in the lower layers of the latter, the lower Washington Formation [17], implying an earlier age, possibly Asselian. Baars [18,19] also supported an earlier age of the Cutler Group in southwest Colorado, suggesting equivalence with the Halgaito Tongue and lower Supai Formation of Utah and lower Abo Formation of New Mexico. These formations are considered earliest Cisuralian (Asselian-Sakmarian) or possibly latest Carboniferous in the case of the Halgaito Tongue [16]. Since MCZ 2985 was found in the uppermost 200ms of the section, an Asselian-Sakmarian age seems reasonable, but it should be noted that these correlations are mainly based on tetrapod biostratigraphy.

### Systematic Palaeontology

**Synapsida** Osborn, 1903 [20]

**Caseasauria** Williston, 1912 [21]

Family **Eothyrididae** Romer & Price, 1940 [1]

Genus ***Vaughnictis*** gen. nov.

1965 *Mycterosaurus*; Lewis & Vaughn [8]

#### Type species

*Mycterosaurus smithae* Lewis & Vaughn, 1965 [8]

#### Diagnosis

Distinguished from other Eothyrididae by the unusually small temporal fenestra, and concomitantly long post-temporal region, the tall suborbital process of the jugal, and an elongate postfrontal, approximately equal in length to the parietal. The squamosal has a larger anterodorsal process than in *Eothyris*, greatly reducing the postorbital contribution to the dorsal edge of the temporal fenestra. As in *Eothyris*, but differing from *Oedaleops*, the postorbital bar is wide, with extensive overlap between the postorbital and jugal bones. Distinguished from other pelycosaurian-grade synapsids by the considerably greater posterior extension of the posterior ramus of the maxilla, reaching beyond the posterior margin of the temporal fenestra, and the unusually large palatal dentition.

#### Etymology

The first part of the generic name is a homage to Peter Vaughn, who, as co-author of the original publication, completed the original description of the species from this locality, and has made substantial contributions to our knowledge of the Paleozoic vertebrates of North America; -*ictis* refers to the fact that this was a predatory carnivore with stabbing teeth.

***Vaughnictis smithae*** comb. nov.

urn:lsid:zoobank.org:act:CC5F8F0B-F38E-4683-9473-3EDC260791BC

*Mycterosaurus smithae* Lewis & Vaughn, 1965 [8]

#### Type specimen

Holotype: MCZ 2985 a partial skull; a string of six dorsal vertebrae; several ribs; a left femur and tibia; other fragments.

#### Diagnosis

As for genus

#### Type Locality and Horizon

Placerville Locality 11, San Miguel County, Colorado (38.0° N, 108.0° W). Cutler Group, Asselian-Sakmarian.

### Description

**Fig 1:**
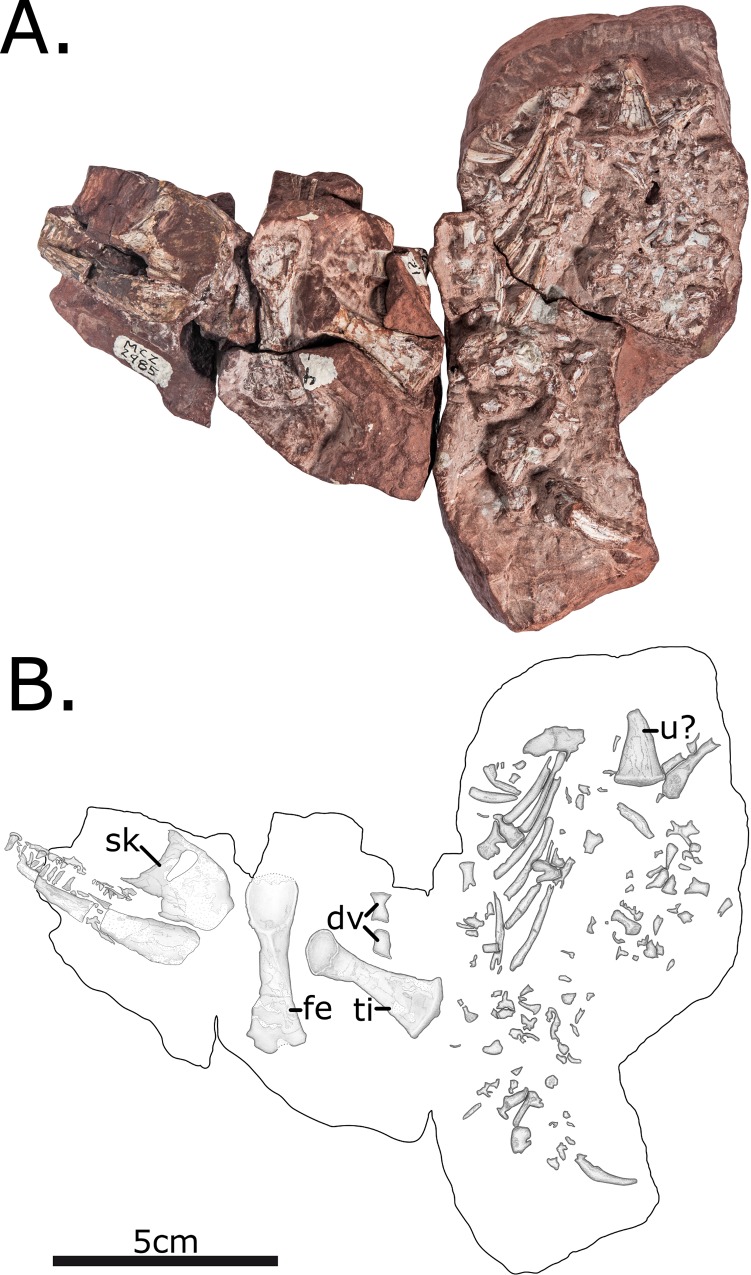
MCZ 2985, after preparation. (A) Photo; (B) Interpretative drawing. Anatomical abbreviations: **dv**–dorsal vertebra; **fe**–femur; **ti**–tibia; **u**–ulna.

MCZ 2985 consists of a previously articulated nodule bearing a skull and several postcranial fragments, including five vertebrae, ribs and a proximal femur and tibia (Fig 1). During the course of the preparation following its original discovery, the skull was separated from the block bearing postcranial material, and the postcranial nodule has been separated into multiple blocks in order to expose the material, although these fragments still articulate.

#### Skull and Lower Jaw

**Fig 2:**
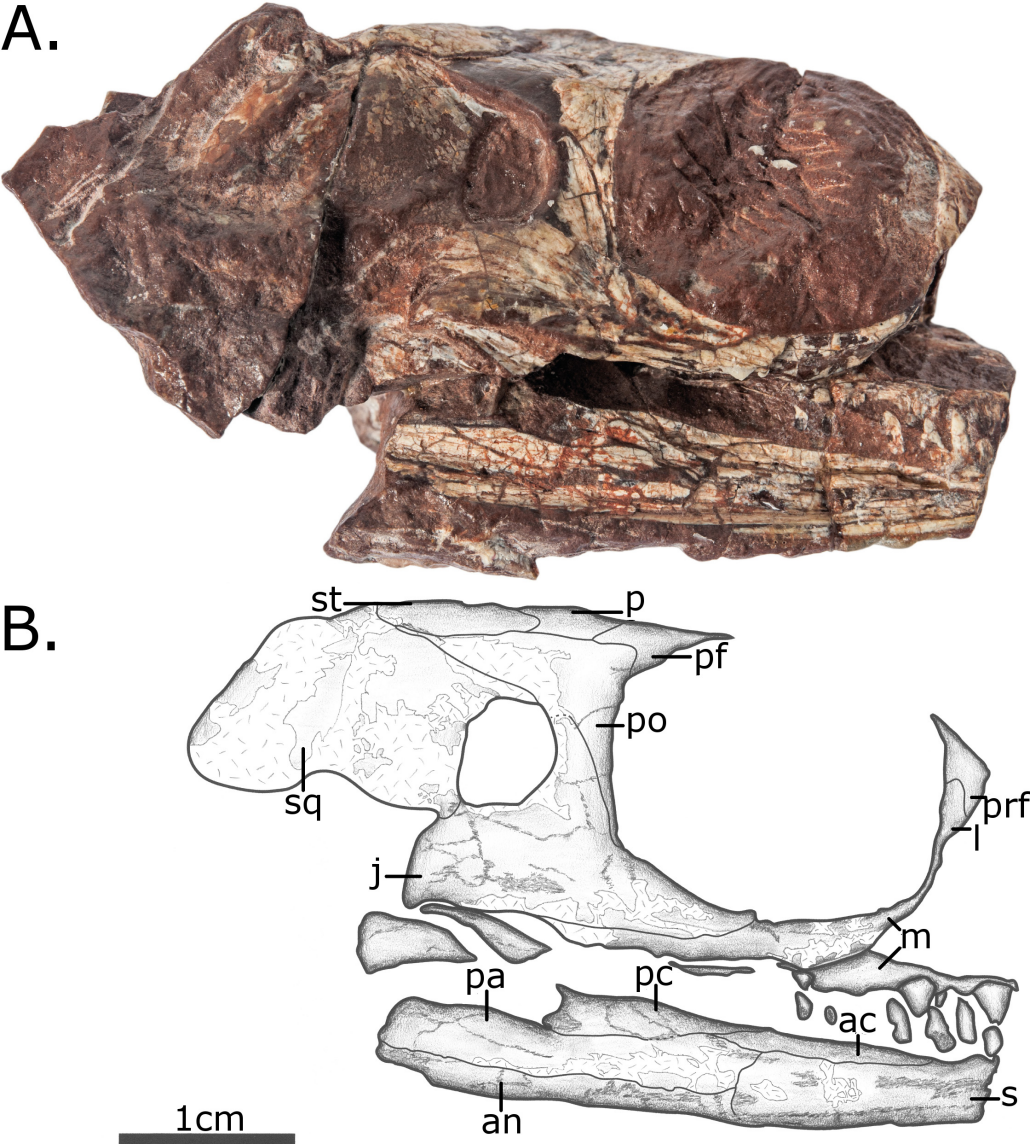
Skull and lower jaw of MCZ 2985 in right lateral view. (A) Photo; (B) Interpretative drawing. Anatomical abbreviations: **ac**–anterior coronoid; **an**–angular; **j**–jugal; **l**–lacrimal; **m**–maxilla; **p**–parietal; **pa**–prearticular; **pc**–posterior coronoid; **pf**–postfrontal; **po**–postorbital; **prf**–prefrontal; **sp**–splenial; **sq**–squamosal; **st**–supratemporal.

**Fig 3:**
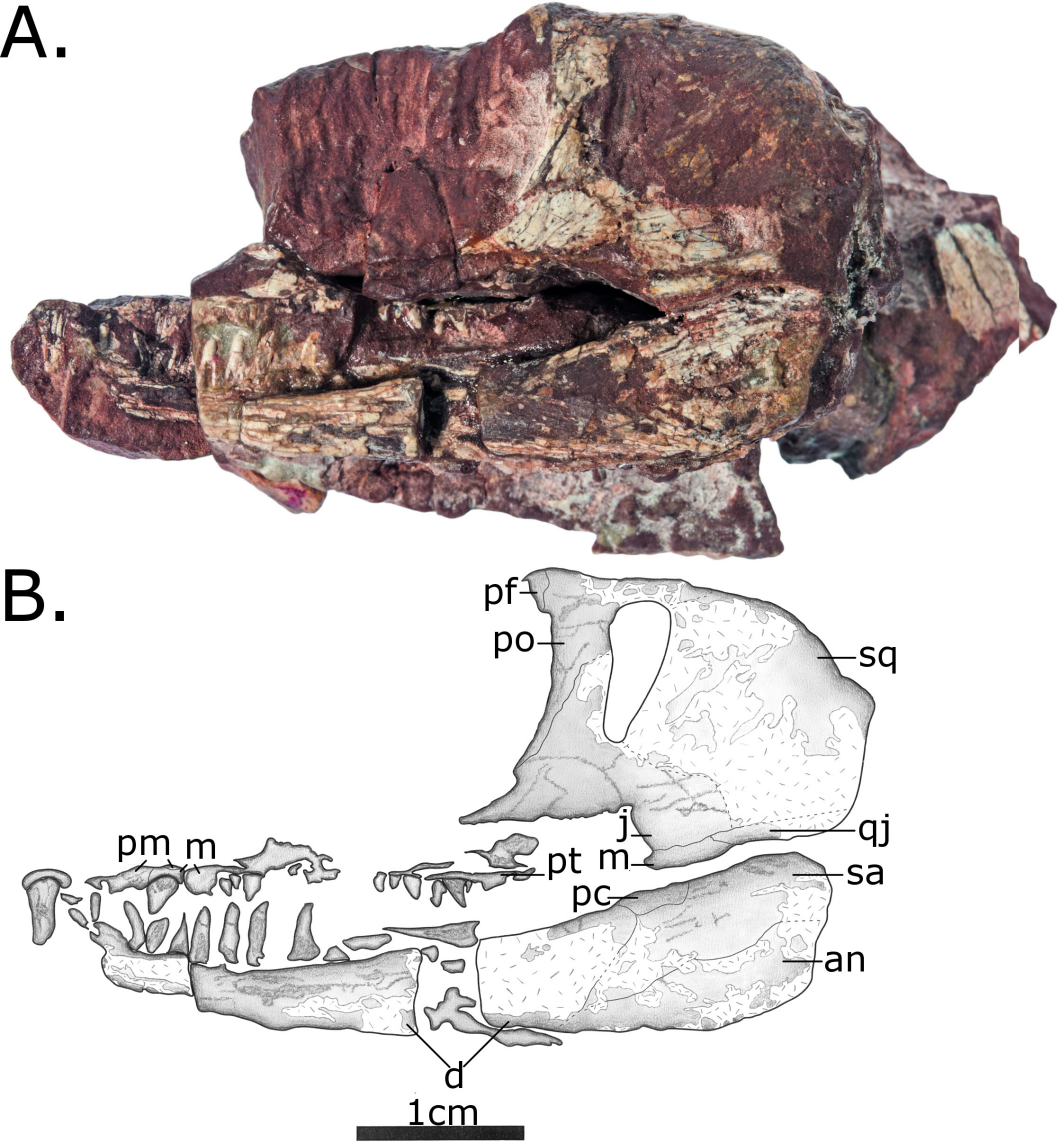
Skull and lower jaw of MCZ 2985 in left lateral view. (A) Photo; (B) Interpretative drawing. The premaxilla, maxilla and tip of the dentary is a separate fragment articulating with the skull, and represents an internal view of the right upper and lower jaw fragments preserved on a counterpart. Anatomical abbreviations: **an**–angular; **d**–dentary; **j**–jugal; **m**–maxilla; **pc**–posterior coronoid; **pf**–postfrontal; **pm**–premaxilla; **po**–postorbital; **qj**—quadratojugal **sa**–surangular; **sq**–squamosal.

**Fig 4:**
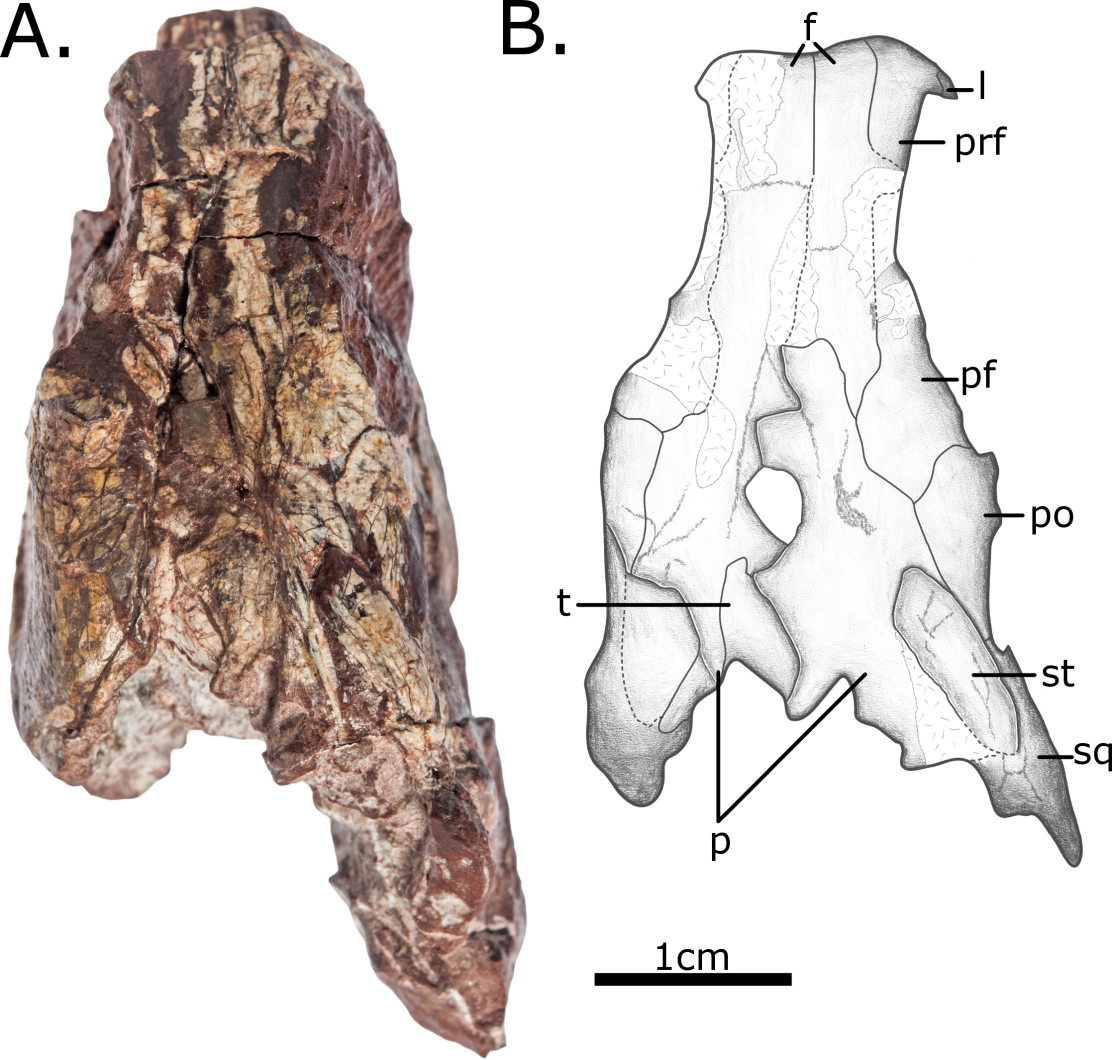
Skull of MCZ 2985 in dorsal view. (A) Photo; (B) Interpretative drawing. Anatomical abbreviations: **f**–frontal; **l**–lacrimal **p**–parietal; **pf**–postfrontal; **po**–postorbital; **prf**–prefrontal; **sa**–surangular; **sq**–squamosal; **st**–supratemporal.

The skull (Figs 2–4) is preserved in three dimensions, although slightly compressed laterally and distorted, being sheared dextrally along the vertical plane through its long axis. The preservation quality of the skull roof hampers the clear identification of sutures. The sutures on the lateral surfaces of the skull are considerably easier to determine, particularly on the right side (Fig 2). Most of the occiput and palate is not exposed. The synchrotron scan did reveal the palate, although the bones were too fractured (presumably by the lateral compression of the skull) to reliably ascertain the sutures. The orbit is relatively large, whereas the temporal fenestra is unusually small compared to other pelycosaurian-grade taxa, less than a quarter of the length of the orbit. The shape of the temporal fenestra appears to be substantially different on the two sides, suggesting that the original preparation may have damaged the left side, and so the right fenestra is considered closer to the true morphology (see S1 Fig for a closer view of the damaged left temporal fenestra). The fenestra is oblong in shape, rather than being narrower ventrally as in ophiacodontids or dorsally as in most other pelycosaurian-grade synapsids, including *Oedaleops*. Its shape is instead more similar to that of *Eothyris* and some mycterosaurine varanopids [5,22,23]. Its dorsoventral height is slightly greater than its anteroposterior length.

The antorbital region is missing except for a separate fragment that preserves part of the left maxilla and premaxilla with teeth as well an internal view of the tip of the right mandible (Fig 3). Only a small part of the premaxilla is preserved in medial view on this small separate fragment, which fits as a counterpart and extends the anteriormost preserved part of the skull. No useful information about premaxilla can be discerned, except for some aspects of its dentition (see **Dentition** below). The septomaxillae and the nasals are not preserved.

The bones of the skull roof are heavily eroded, although what is preserved does not show any evidence of the ornamentation found in caseids. The frontal is the anteriormost preserved element of the skull roof. Its anterior margin is not preserved, so it is not possible to ascertain its original length. The bones around the dorsal margin of the orbits are damaged, but it appears likely that a small lateral lappet of the frontal contacted the orbit. The posterior process of the frontal is only visible on the right side, and forms a short wedge-shaped process between the parietal and the postfrontal, but still leaving a substantial contact between the two, unlike in varanopids and ophiacodontids where the contact is limited.

The parietals have been displaced so that the posterior end of the right element overlies that of the left. Despite this, one can see that the pineal foramen is large and positioned at about midlength of the parietal, as in other eothyridids.Only fragments of the maxilla are preserved, better on the right than the left side. The posterior process of the maxilla is a narrow splint extending beyond the level of the temporal fenestra, and contributes to the lower orbital margin.

A fragment of the right lacrimal is preserved at the anterior edge of the orbit. A ventral process of the prefrontal incises the lacrimal and limits its contribution to the orbital margin in lateral view, a feature of sphenacodontids and some varanopids. A lacrimal foramen cannot be identified.

The lateral surface of the prefrontal is flat, lacking the concavity observed in sphenacodontids and ophiacodontids. In dorsal view the prefrontal has a long, narrow posterior process forming about a third of the upper margin of the orbit.

The postfrontal, best preserved on the right side, is an unusually long, transversely narrow triangular element with a flat dorsal surface. It contacts the parietal posteriorly but is separated from it anteriorly by the posterior process of the frontal. The posterior margin of the postfrontal is incised by an anterior protrusion of the postorbital, a feature shared with *Eothyris* and sphenacodontids.

The postorbital is a robust element with a broad posterior process. It has a posterior contact with a squamosal, but this contact does not extend far back over the temporal region as in mycterosaurine varanopids and some sphenacodontids. The ventral process of the postorbital and the dorsal process of the jugal form a relatively thick postorbital bar, similar to those of *Eothyris* and *Eocasea* [5,24].

The jugals are also robust elements: both the anterior and posterior rami are dorsoventrally thick. The anterior ramus is short, not reaching anterior to the orbital midline, but the posterior ramus extends well beyond the posterior margin of the temporal fenestra reaching at least halfway along the post-temporal region (the erosion of the lateral surface of this region makes identifying the full extent impossible).

The supratemporal is preserved on the right, but eroded away on the left. It is a large element set in a depression in the parietal, more similar in proportions to that of caseasaurs than to the splint of bone seen in varanopids and sphenacodontids. It is oblong in shape.

The squamosal is broad, flat and has a lateral exposure similar to that seen in ophiacodontids: the length of the posttemporal region is considerably greater than the breadth of the temporal fenestra. The temporal fenestra itself is bordered anteriorly by the jugal and dorsally by the postorbital and squamosal; there is no anterior process of the squamosal contacting the jugal dorsally.

On both sides of the skull a narrow splint of bone excludes the jugal from the ventral margin of the skull, formed from both the posterior process of the maxilla and the anterior process of the quadratojugal. The contribution of the quadratojugal to the exclusion of the jugal to the ventral margin of the skull, visible on the left side (Fig 4) is reduced relative to other caseasaurs, wherein the anterior ramus reaches anteriorly beyond the temporal fenestra [5,25]. *Vaughnictis* shows the condition found in mycterosaurine and varanodontine varanopids with the posterior ramus of the maxilla having the greatest contribution [21,22]. In *Vaughnictis*, in fact, the posterior ramus of the maxilla extends further posteriorly than in any observed pelycosaurian-grade synapsid, reaching beyond the posterior margin of the temporal fenestra.

The occipital and ventral sides of the skull of MCZ 2985 are almost entirely covered by matrix. However, a small portion of the pterygoid is exposed in left lateral view between the left maxilla and mandible. Not much can be said about the morphology of the pterygoid, but it bears teeth (see **Dentition** below).

#### Mandible

Both left and right mandibles are preserved, the right as a counterpart showing the medial sutures. The tip of the left mandible is missing, but that of the right is preserved on the separate fragment also bearing the maxilla and premaxilla. The mandible is a gracile element with slight curvature, narrowing anteriorly. The coronoid eminence is only a slight prominence, positioned more posteriorly than that of *Eothyris* [5]. It is formed laterally by the posterior coronoid.

The dentary is the largest element in lateral view, although it does not quite reach two thirds of the length of the mandible. The splenial does not appear to have the lateral exposure seen in caseids, edaphosaurids and sphenacodontids. On the medial surface, the splenial covers about half the length of the mandible, not reaching posteriorly enough to contact the posterior coronoid. The angular is visible in both lateral and medial views. On the lingual side it extends anteriorly about halfway along the length of the mandible. There is no keel on its ventral surface. The prearticular covers a similar length.

#### Dentition

One premaxillary tooth is preserved with space for two more behind it. This tooth is enlarged relative to the maxillary teeth, implying that it is the anteriormost tooth (this tooth is also enlarged in *Eothyris*).

Four maxillary teeth are preserved on each side of the skull. Two of those preserved on the right side are larger and more robust than the others. From their position (under the anterior margin of the orbit) it might be suggested that these represent the secondary caniniform region seen in *Eothyris* and *Oedaleops*. The maxillary teeth are conical, with no serrations and only slight recurvature.

**Fig 5:**
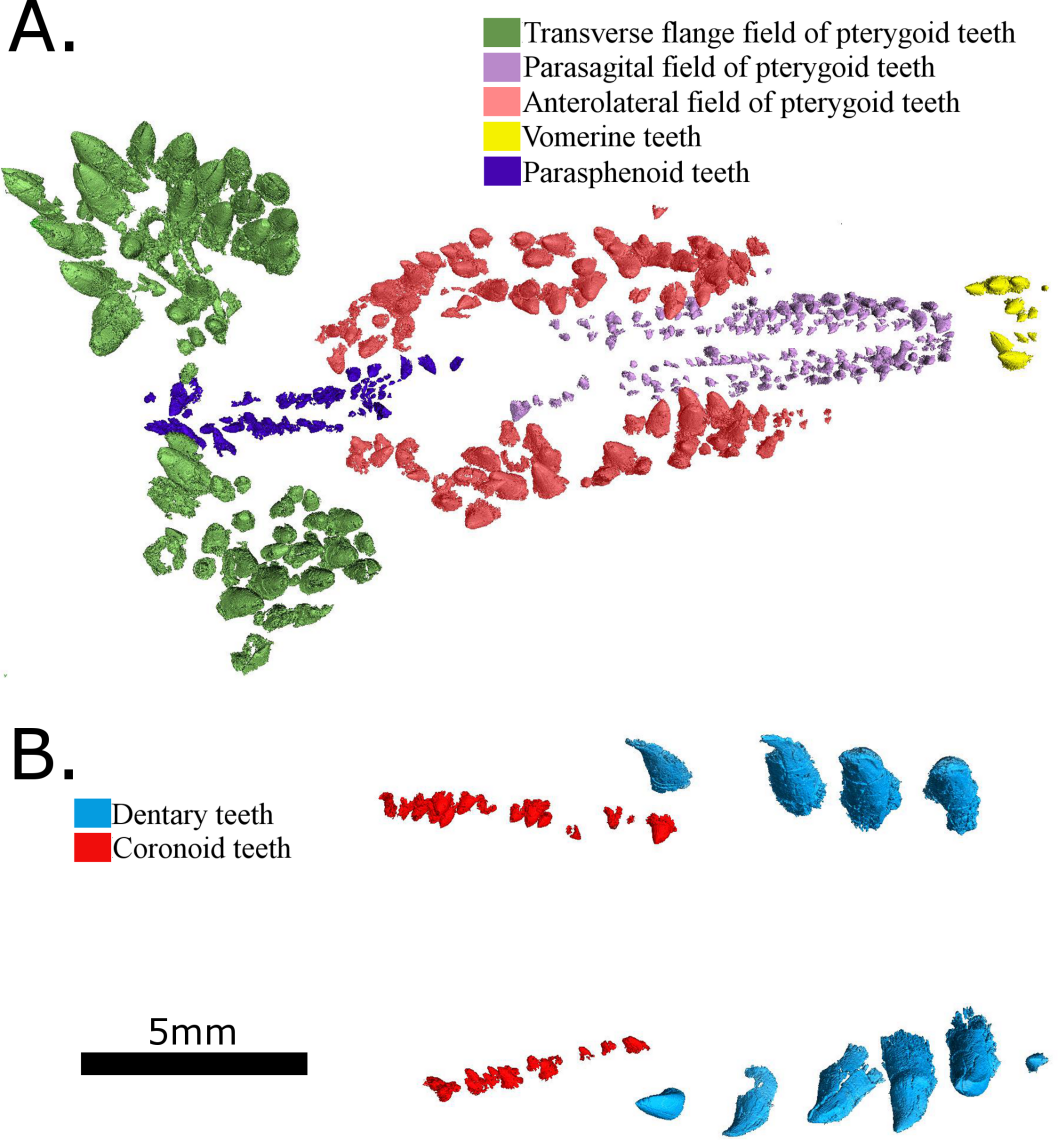
Reconstructions of the dentition of MCZ 2985 based on synchrotron scans. (A) The palatal dentition (ventral view); (B) The mandibular dentition (dorsal view).

Lewis and Vaughn [8] mentioned the pterygoid teeth visible in the left lateral view of the skull, but were unable to ascertain anything other than their large size. The synchrotron scan of the specimen has now allowed the morphology and arrangement of the palatal dentition to be studied in more detail (Fig 5A).

The cultriform process of the parasphenoid possesses two rows of small teeth running along its entire length. Among synapsids, teeth on the cultriform process is only known in caseasaurs (Stovall 1937, Sigogneau & Russell 1979, Maddin et al. 2008) and varanopids (Berman & Reisz 2001), in which they seem to have evolved independently. Parasphenoid teeth are also known in a number of other basal amniote groups e.g. millerettids [26], lanthanosuchoids [27], captorhinids [28] and basal diapsids [29], but do not appear to be a plesiomorphic feature of amniotes. The region of the parasphenoid posterior to the parabasisphenoid tubera does not appear to be preserved, and it is unknown if the ventral plate bore palatal teeth, as found in the caseid *Euromycter* [30]. The poor quality of preservation of the palatal surface of *Eothyris* does not allow us to determine the extent to which the parasphenoid in that taxon had teeth.

The pterygoid teeth are arranged in three distinct fields: one on the transverse flange, a diagonal second field that is continued anterolaterally on the palatine, and a third parasagittal field that extends anteriorly. The presence of these three distinct fields of teeth likely represents the primitive condition for caseasaurs, being found in the caseids *Cotylorhynchus romeri* and *Euromycter rutenus* [30,31], but may also represent a more basal, amniote synapomorphy. The teeth on the transverse flanges are unusually large, only slightly smaller than the maxillary teeth, with the largest teeth being located along the posterior edge of the flange. This probably represents an autapomorphy of *Vaughnictis*, as is the unusually large size of the tooth field that appears to cover the entire transverse flange. It is not possible to determine if some of the teeth on this field extended onto the ectopterygoid, as in some reptiles.

The two other fields of teeth are located on the anterior process of each pterygoid, The field of larger teeth extend anterolaterally from the region of the interpterygoid vacuities, and most likely extends onto the palatine bones anteriorly. The position of this field relatively close to the midline of the skull is most likely the result of distortion of the skull, with the skull being compressed transversely and the palate having been vaulted. In other caseasaurs and in basal reptiles this field is angled more steeply anterolaterally [25,30]. The third field, composed of teeth that are similar in size to those on the cultriform process, extends anteriorly on either side of the midline pterygoid suture. Since we are uncertain of the suture between the vomer and pterygoid, it is not possible to determine if this third field of teeth extends onto the vomers, medial to the internal nares, as is seen in basal sphenacodontids and basal reptiles, or whether it was restricted to the pterygoid, as seen in *Cotylorhynchus romeri* and *Ennatosaurus tecton* [25,31].

A field of small teeth is present on the vomer, similar in size and number to those found in *Cotylorhynchus romeri*, but instead of a single row, there are two pairs of rows on either side of the midline. The vomerine teeth of the lateral row are larger than those of the medial row. Overall, the general impression provided by the palatal surface is that its dentition is that of a small predator with extensive fields of teeth that are designed to pierce and hold its prey.

In the lower jaws, four teeth are preserved on the right and five on the left dentary. Another five poorly preserved teeth are visible in the dentary tip on the separate fragment. These are uniform in size and identical in morphology to the maxillary teeth: conical and only slightly recurved.

The synchrotron scan has revealed the presence of coronoid teeth (Fig 5B) in this taxon, which are of smaller size than those on the dentary. Their presence is plesiomorphic for synapsids, but they have been lost numerous times, including in derived caseids, ophiacodontids, varanopids and sphenacodontians.

#### Axial skeleton

**Fig 6:**
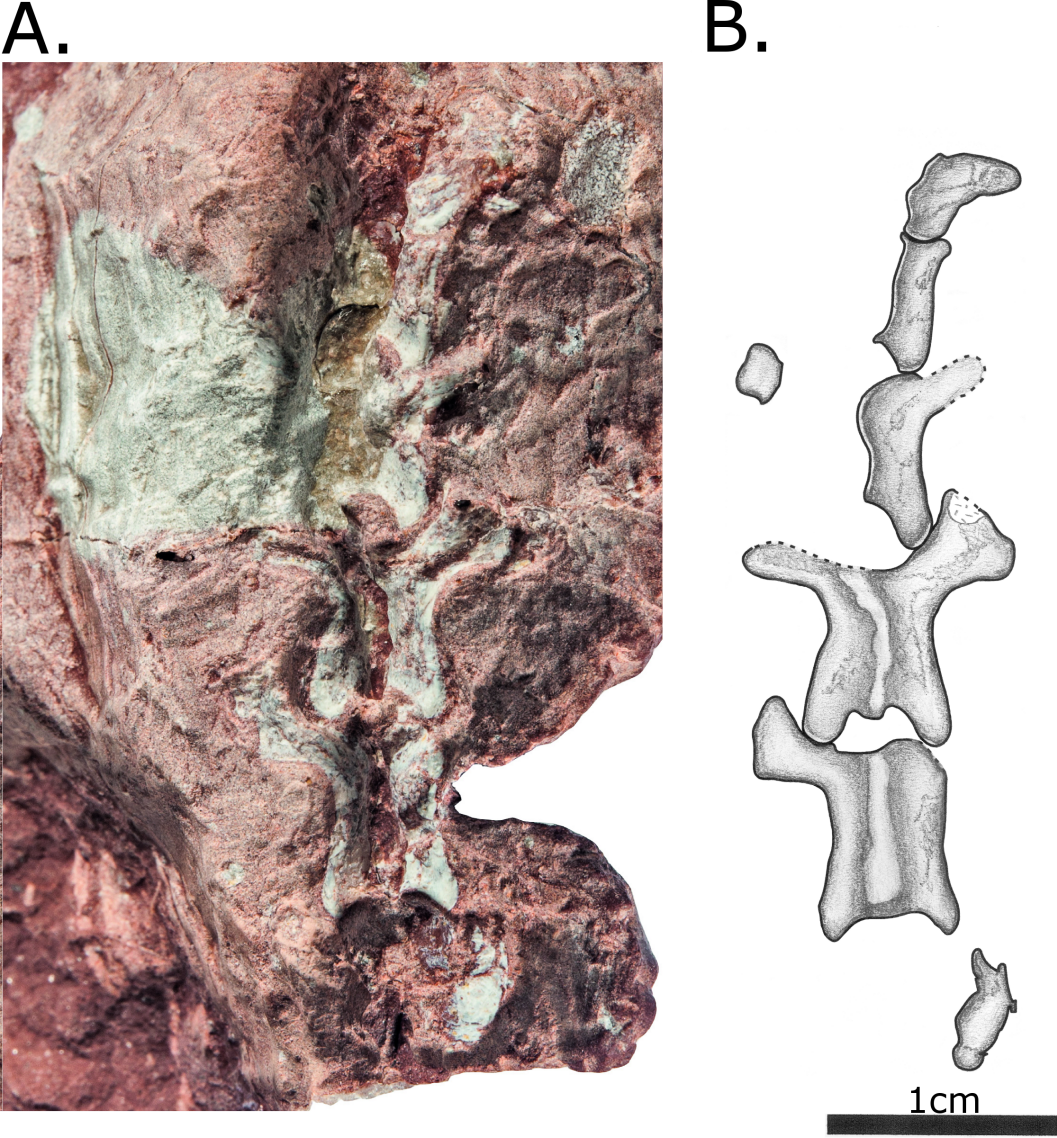
Dorsal vertebrae of MCZ 2985 in dorsal view. (A) Photo; (B) Interpretative drawing.

**Fig 7:**
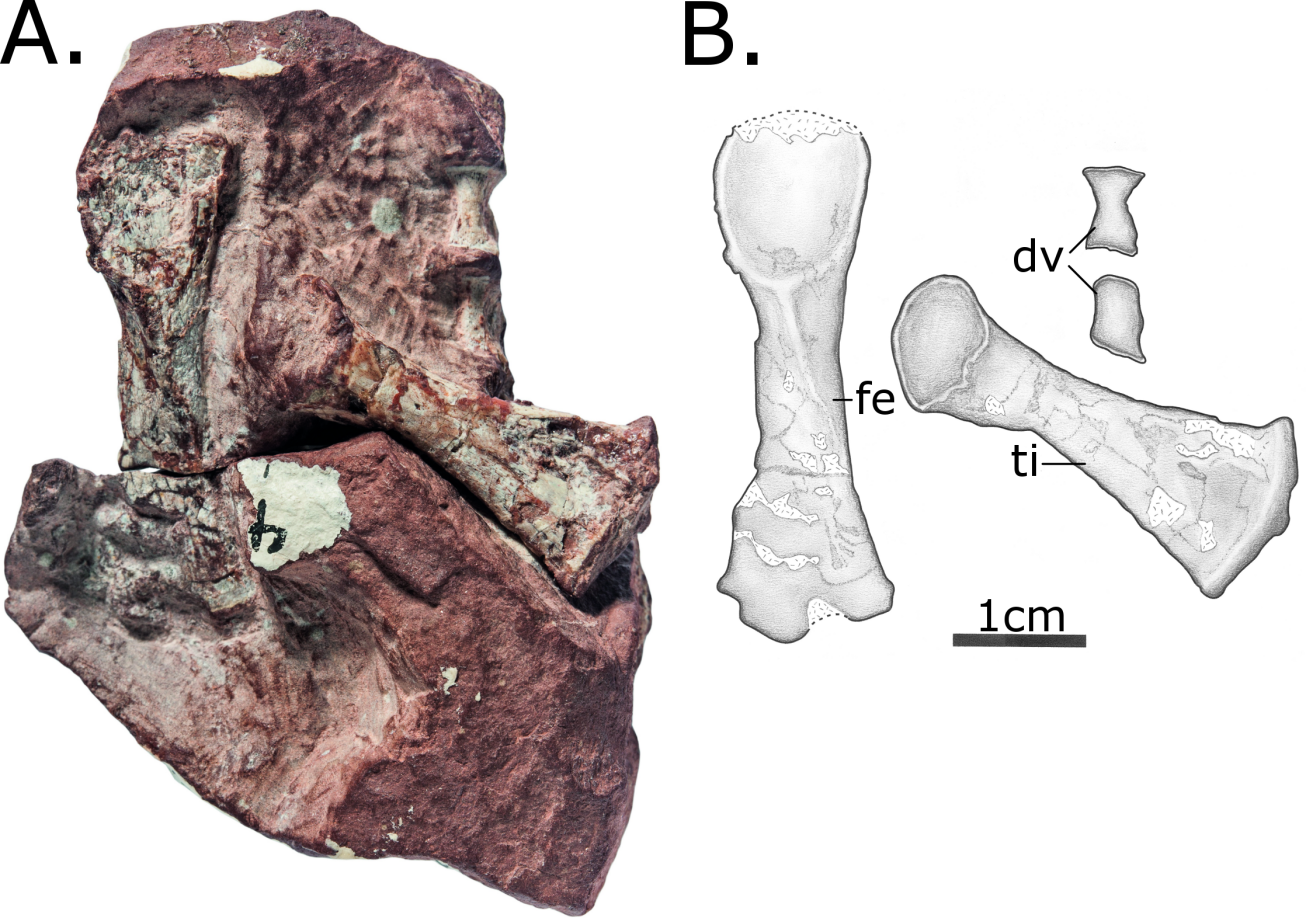
Postcranial material of MCZ 2985. (A) Photo; (B) Interpretative drawing. Includes two dorsal vertebrae, left femur and left tibia, all in ventral view. Anatomical abbreviations: **dv**–dorsal vertebrae; **fe**–femur; **ti**–tibia.

Recent preparation has exposed a series of six dorsal vertebrae in dorsal view (Fig 6), three of which are mostly uninformative. Two of these vertebrae are also exposed ventrally (Fig 7). The vertebrae are plesiomorphic in anatomy, very similar to those described for *Oedaleops* [7]. The ventral surface is rounded, without the keel which is usually present in varanopids and sphenacodontids, and without the pattern of ventral ridge and longitudinal troughs as in ophiacodontids. The neural spines have been slightly eroded dorsally so that no information is available on their exact height, but the bases indicate a blade-like morphology. The neural arches are again plesiomorphic, with no swelling or buttressing. The prezygapophyses show a flat morphology, without the concave surface seen in some caseids. The postzygapophyses are widely spaced with no hyposphene visible. The transverse processes are positioned far anteriorly on the vertebrae, a feature of some caseids and edaphosaurids. They are broad and flat and project slightly anteriorly; whether this is distortion or a feature of their morphology is unclear.

**Fig 8:**
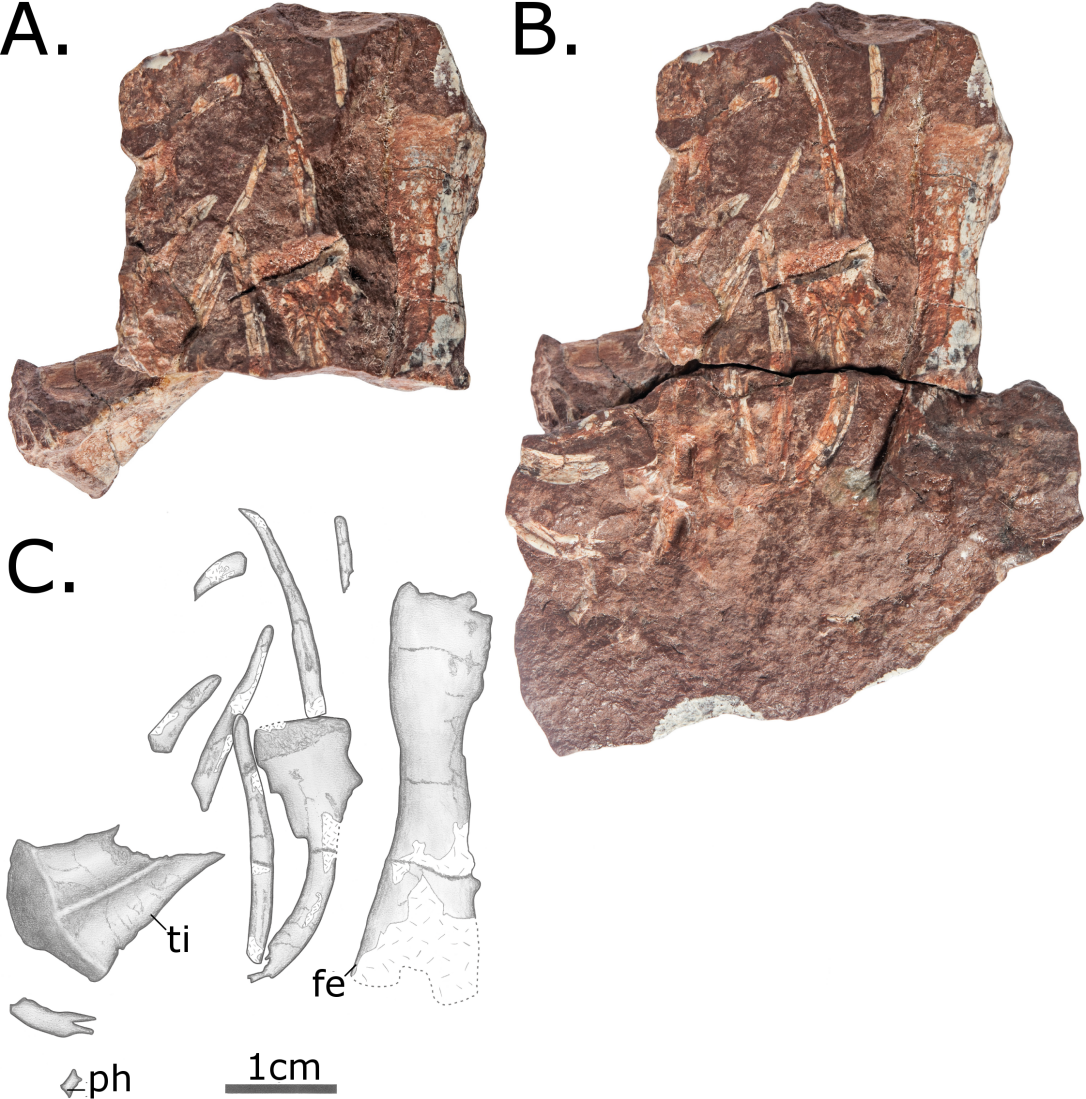
Two articulating rock fragments bearing postcranial material of MCZ 2985. Includes a left femur and tibia in dorsal view, a phalanx of unclear origin and rib fragments. (A) The first of these fragments, fully exposing the proximal part of the left tibia in dorsal view; (B) The two fragments in articulation, covering part of the proximal part of the tibia but showing the almost complete right femur in dorsal view; (C) interpretative drawing incorporating information from both of these views. Anatomical abbreviations: **fe**–femur; **ti**–tibia; **ph**—phalanx.

**Fig 9:**
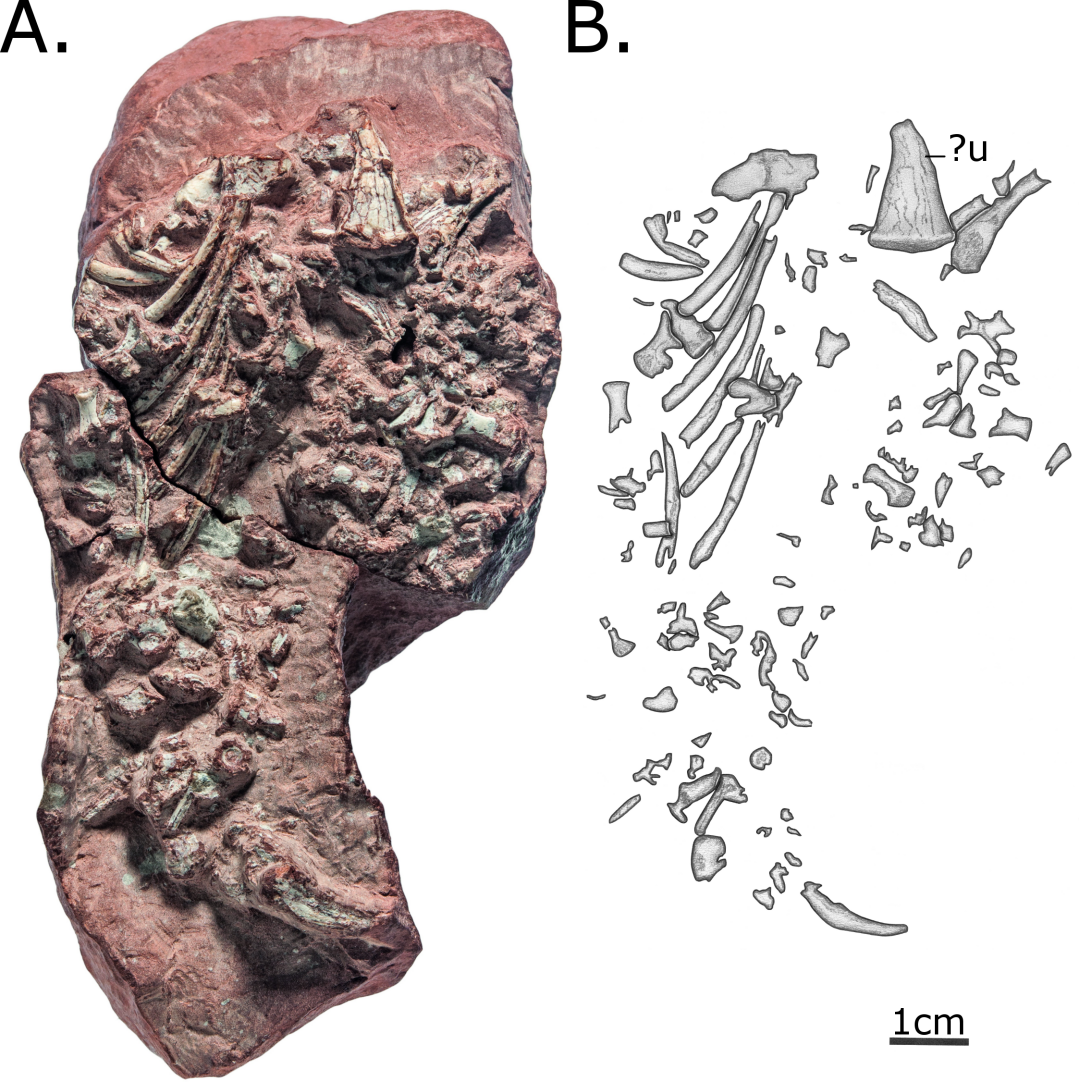
Block with postcranial material of MCZ 2985. (A) Photo; (B) Interpretative drawing. Includes ribs and possible distal ulna. Anatomical abbreviations: **u**–ulna.

Fragments of at least 14 dorsal ribs are preserved on various postcranial blocks (Figs 8 and 9). The ribs are curved only proximally, in contrast to those of herbivorous pelycosaurian-grade synapsids, which are curved throughout their length to form a barrel-like chest. In proportions, however, they appear to be thick relative to the size of the vertebrae, more similar to derived caseids than those of *Oedaleops* and *Eocasea* [7,24].

#### Appendicular skeleton

Recent preparation has revealed the right femur fully in dorsal (Fig 8) and ventral (Fig 7) views. It is very plesiomorphic in its anatomy and agrees in most details with that of *Oedaleops*. However, the femoral shaft of MCZ 2985 is considerably more robust, the proportions being more similar to those of sphenacodontids. The head of the femur is short relative to its total length. The shaft is almost straight, and is oval rather than circular in cross section. The internal trochanter has been eroded away, but a prominent fourth trochanter is preserved. The internal fossa is enclosed posteriorly by a ventral ridge, this plesiomorphic condition being absent in caseids. A low longitudinal ridge extends proximodistally across the ventral surface of the femur. There is no longitudinal mound on the proximodorsal surface, a condition independently evolved in several clades but unfortunately not visible in *Oedaleops*. The distal end of the femur is damaged, but some valuable information is available. The condyles are separated with virtually no difference in distal expansion, as in *Oedaleops* and *Eocasea* [7,24] but unlike in more derived caseids. Also similar to *Oedaleops* and *Eocasea* but different to more derived caseids is the lack of compression of the anterior condyle. The dorsal surface of the posterior condyle is not concave as in some varanopids.

The right tibia has been exposed completely in ventral view (Fig 8), whereas only the proximal part is visible in dorsal view (Fig 9A). Like the femur, its morphology is very similar to *Oedaleops* [7]. It is almost straight with a low ridge along the ventral surface and a prominent cnemial crest. However, the proportions of the tibia of *Vaughnichtis* differ from that of *Oedaleops* in that it is shorter but considerably thicker throughout.

### Eothyridid Affinities of MCZ 2985

*‘Mycterosaurus’ smithae* has received only limited attention in the literature after its original description. Berman and Reisz [22], in their re-description of the type species of *Mycterosaurus*, *M*. *longiceps*, tentatively kept *M*. *smithae* within Varanopidae, though noting that its assignment to the genus appeared to be based largely on a process of elimination of all other known genera of comparable size. Reisz [3], in his thorough review of pelycosaurian-grade synapsid species undertaken for the Handbook of Paleoherpetology, also noted the lack of varanopid synapomorphies in *‘M’*. *smithae*, and suggested the assignment should be considered provisional.

This detailed re-examination of MCZ 2985, including new features revealed in preparation and synchrotron scanning, allows the rejection of the affinity of this species with *Mycterosaurus*, Mycterosaurinae or Varanopidae. *Vaughnictis* has a ventral ridge system on the femur, the lack of which was considered characteristic of *Mycterosaurus* [22]. Most unambiguous varanopid and mycterosaurine synapomorphies that could be compared reject a varanopid affinity for MCZ 2985: the femur is not slender, the lateral dentition is not serrated or strongly recurved, teeth are present on the coronoid and there is no lateral boss on the postorbital.

The large supratemporal is set in the parietal and in contact with the postorbital, supporting the assignment of *Vaughnictis* to Caseasauria, the clade containing the families Caseidae and Eothyrididae. Further evidence for this assignment includes the short dentary relative to the rest of the lower jaw and the large size of the pineal foramen. An affinity with Eothyrididae is suggested based on the extension of the posterior ramus of the jugal beyond the temporal fenestra and the position of the pineal foramen midway along the midline of the parietal. Eothyrididae thus far contains only two species of small carnivorous basal synapsids: *Eothyris parkeyi* and *Oedaleops campi*. Of the two, *Vaughnictis* is most similar to *Eothyris*, sharing with this species the thick postorbital bar, the incision of the postfrontal by the postorbital and the small temporal fenestra with an oblong rather than trapezoid or triangular shape. The separate fragment bearing the dentary tip, premaxilla and maxilla indicate a large overbite, another similarity with *Eothyris*.

It is unlikely that *Eothyris parkeyi* and *Vaughnictis* represent two specimens of the same species; *Vaughnictis* has a shorter posterior process of the postorbital, a more posteriorly placed coronoid eminence, a smaller temporal fenestra and a greatly expanded lateral surface of the squamosal (more similar in extent to Ophiacodontidae). Perhaps the most striking feature of *Vaughnictis* is the massive dentition on the palate, the unusually large size of the teeth on the transverse flange, and the extensive dentition on the cultriform process of the parasphenoid. There could, meanwhile, be some justification for assigning them to the same genus. *Vaughnictis* shares with *Eothyris* the contribution of the maxilla to the margin of the orbit, a character Reisz et al. [5] considered diagnostic for *Eothyris*. However, this is a highly variable character, also seen in some derived caseids and varanopids. Other autapomorphies of *Eothyris* could not be compared with the less complete skull of *Vaughnictis*. Therefore, in this study it is considered more conservative to regard them as separate genera.

Eothyrididae is widely considered to consist of small, agile carnivores or insectivores [1,2,5]. The recent description of new material of *Oedaleops campi* [7], including the first eothyridid postcranium, supports this ecological hypothesis, illustrating generalised tooth structure, long gracile limbs and ribs with only proximal curvature, unlike those of herbivorous caseids. The mandibular dentition of *Vaughnictis* is of the same conical shape, with little recurvature and no visible serrations, possibly indicating a similar diet. Unfortunately, very little information is available on the upper dentition; the region where the robust caniniform teeth are seen in *Eothyris* is not preserved, and the presence of a secondary caniniform region cannot be confirmed with certainty. The available information suggests that *Eothyris* did not possess the massive palatal dentition of *Vaughnictis*, but the palate is unknown in *Oedaleops*. An important difference between *Oedaleops* and *Vaughnictis* is in the robusticity of the limbs and the skull; although *Oedaleops* has a longer femur and tibia, those of *Vaughnictis* are considerably broader, both at the distal ends and at the mid-shaft (Table 1). The postorbital and subtemporal bars are also thicker than their equivalents in *Oedaleops*. It appears that *Vaughnictis* may not have led the lifestyle of an agile insectivore that is inferred for *Oedaleops*, but may have been a more robust carnivore. A durophagous diet might even be indicated; the dense covering of palatal dentition might have acted as a “tooth plate”. Alternatively, it is possible that the differences in proportions were ontogenetic; the small size and large orbit of *Vaughnictis* could indicate a young individual. However the state of ossification of the limb bones suggests otherwise; all condyles and trochanters of the femur and the cnemial crest of the tibia are well ossified. Extensive palatal dentition is also uncharacteristic in juveniles of modern reptiles [32,33], although the dental ontogeny of pelycosaurs has at present not been studied in detail Other explanations must be sought for the unusual morphology of *Vaughnictis*. It is clear from this specimen that there was a greater morphological diversity and potentially greater ecological diversity within eothyridids than has previously been suspected.

**Table 1:** Postcranial measurments of *Vaughnictis smithae* compared to other early synapsids.

	*Vaughnictis smithae*	*Oedaleops campi*	*Eocasea martini*	*Casea broilii*	*Mycterosaurus longceps*	*Archaeovenator hamiltonenesis*	*Varanops brevirostris*	*Ophiacodon retroversus*	*Dimetrodon limbatus*
Dorsal centrum length	6.82	15.64	3.08	30.27	10.23	4.46	18.11	27.97	26.84
Dorsal centrum diameter	4.33	11.07	3.25	36.47	7.8	2.76	14.87	34.62	31.92
Femur length	36.81	45.29	15.02	78.66	59.09	37.20	106.58	176.05	200.02
Femur proximal width	12.08	14.98	?	22.07	16.02	8.49	38.17	69.27	62.34
Femur distal width	14.41	14.1	6.04	24.00	11.31	10.00	40.49	77.2	59.92
Femur mid-shaft width	6.46	5.34	2.47	10.74	6.60	3.35	15.63	29.97	29.14
Tibia length	33.37	50.78	10.70	58.64	47.00	18.95	119.13	144.79	160.62
Tibia proximal width	15.37	11.55	3.91	22.93	8.00	2.88	42.73	51.00	62.73
Tibia distal width	10.37	9.18	2.13	15.51	11.00	2.58	24.68	40.88	38.85
Tibia mid-shaft width	6.38	4.16	1.64	8.1	4.38	1.57	14.6	16.61	20.13

All measurements in mm.

### Comparison with USNM 22098

USNM 22098 is another specimen found at Placerville, although much lower in the section than MCZ 2985, the holotype of *Vaughnictis* [8]. This specimen consists of seven poorly preserved vertebrae, a proximal right femur and shaft, and other undiagnostic fragments. Lewis & Vaughn [8] concluded that this was a second specimen of ‘*Mycterosaurus’ smithae*, although they acknowledged that the preservation was too poor to properly compare it with the type. Their justification for assigning these two to the same species was the similar size of the vertebrae and the occurrence in the same formation and area. They also favourably compared the proportions of the femur of USNM 22098 with that of *Mycterosaurus longiceps*.

Additional preparation of MCZ 2985 has exposed the complete femur, permitting comparison between these two specimens (USNM 22098 shown in S2 Fig). It is clear that they cannot be assigned to the same species. The femur of MCZ 2985 is considerably more robust, with a much thicker shaft than that of USNM 22098. Moreover, the head of USNM 22098 is proportionately much longer relative to its width than that of MCZ 2985. The gracile nature of the USNM 22098 femur hints at a varanopid affinity, although the preservation and current state of preparation of this specimen prevent reliable assignment. Unfortunately no diagnostic characters are visible on the vertebrae.

## The Phylogeny of Pelycosaurian-Grade Synapsids

The phylogenetic position of *Vaughnictis* was assessed using a modified version of the matrix of Reisz & Fröbisch [24]. This was itself based on that of Benson [6], the first global analysis of the relationships of pelycosaurian-grade synapsids (the only change made by Reisz & Fröbisch was the addition of *Eocasea*, the basalmost caseid from the Late Pennsylvanian Hamilton Quarry of Kansas). *Vaughnictis* was added to the matrix, along with four other taxa: *Datheosaurus macrous* and *Callibrachion gaudryi*, two taxa formerly considered sphenacodontian synapsids [1], but recently reassigned to Caseasauria [34]; *‘Casea’ nicholsi*, a caseid from the uppermost Cisuralian Vale Formation of Texas [35]; and *Apsisaurus witteri*, a small early Permian carnivore originally described as a diapsid [36] but reassigned to Varanopidae [37]. Also added to the matrix was the postcranial information now available for *Oedaleops campi* [7]. A number of modifications to the data matrix of Benson [6] were made and five new characters were added (see S1 Appendix for full details of the modifications made to the original matrix). *Basicranodon fortsillensis* was removed from the analysis, following Reisz et al. [38] and Maddin et al. [11], who considered this species a junior synonym of *Mycterosaurus*.

The matrix was analysed with parsimony in the Willi Henig Society edition of TNT version 1.5 [39]. The new technology driven search at level 100 was used, incorporating the drift, sectorial search and fusion algorithms. The minimum tree length was searched for 100 times, followed by a search with the branch swapping algorithm using each of the most parsimonious trees (MPTs) found previously as a starting point. *Limnoscelis* was set as the outgroup. The analysis identified 540 MPTs, with a length of 785, a retention index of 0.74 and a consistency index of 0.43. Support values for individual nodes were calculated using relative fit difference [40].

**Fig 10:**
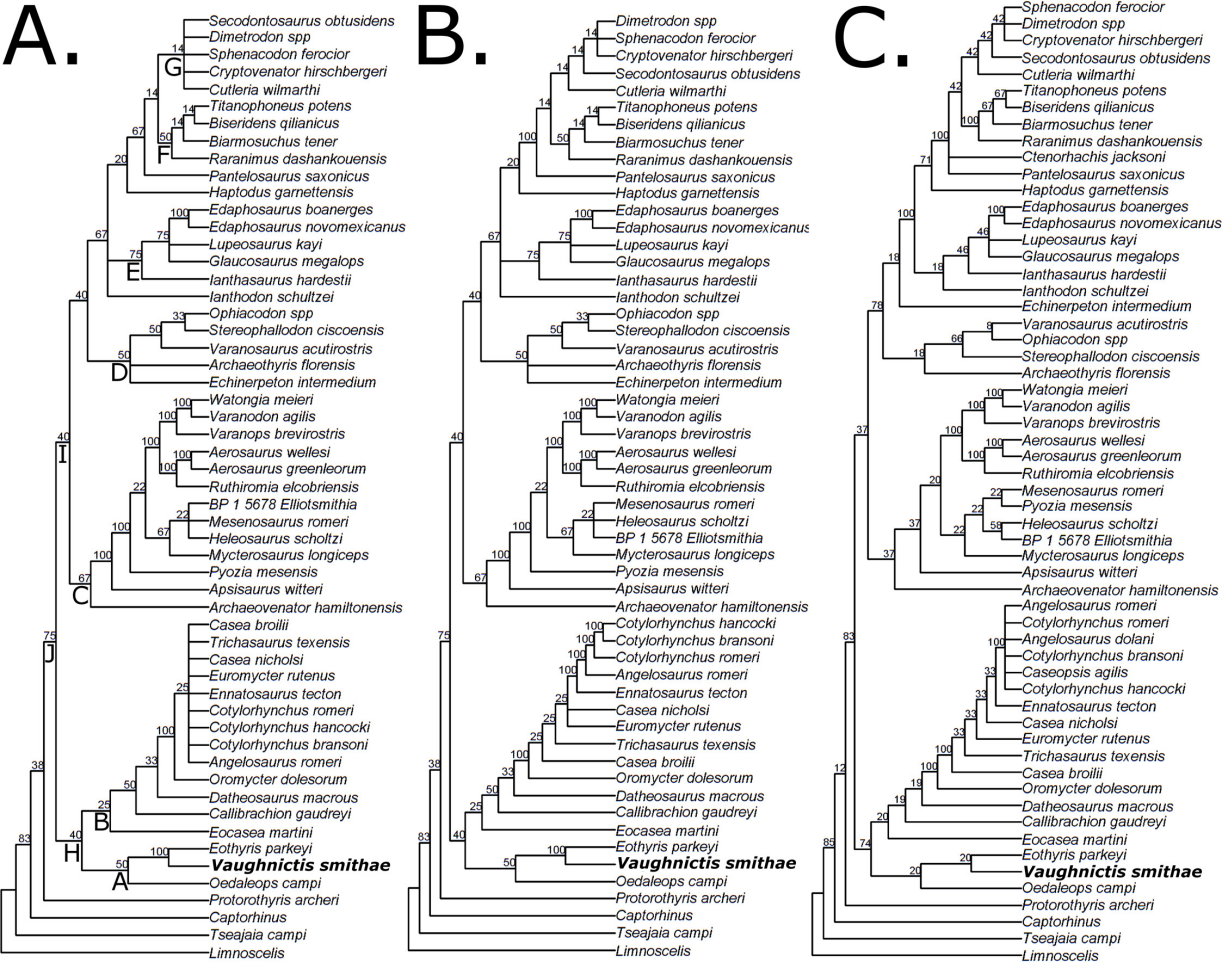
Results of phylogenetic analysis of data matrix using parsimony. (A) Strict consensus of all most parsimonious trees; (B) reduced consensus after removal of the three wildcard taxa; (C) strict consensus of all trees found using implied weights. Numbers at nodes represent support values (relative fit difference). Capital letters at nodes are to label the major clades. **A**–Eothyrididae; **B**–Caseidae; **C**–Varanopidae; **D**–Ophiacodontidae; **E**–Edaphosauridae; **F**–Therapsida; **G**–Sphenacodontidae; **H**–Caseasauria; **I**–Eupelycosauria; **J**–Synapsida.

In all most parsimonious trees, *Vaughnictis* was found to be the sister taxon of *Eothyris parkeyi*, with *Oedaleops campi* the sister to these two eothyridids (Fig 10A). The monophyly of the Eothyrididae was fairly poorly supported, but the sister-taxon relationship between *Vaughnictis* and *Eothyris* received better support.

Relationships elsewhere in the phylogeny were identical to those found in the original analysis by Benson [6], with two exceptions. In the original analysis *Echinerpeton intermedium* was found in three equally parsimonious positions: the basalmost synapsid, an ophiacodontid and the sister to a clade containing caseasaurs, edaphosaurids and sphenacodontians. Here it is found to be an ophiacodontid, based on the supracanine buttress on the medial surface of the maxilla, the short anteroposterior length of the dorsal centra relative to their height and the extensive ventral webbing on the transverse processes of the dorsal vertebrae.

The second difference between the results presented here and those found by Benson [6] is the position of Caseasauria (the clade containing Eothyrididae and Caseidae). Since the review of Reisz [3], caseasaurs have been considered the sister clade to all other synapsids (the Eupelycosauria), a position based primarily on cranial characters. Benson [6] introduced a larger number of postcranial characters into the analysis and found caseasaurs to be more derived, the sister to Sphenacomorpha (the clade containing edaphosaurids and sphenacodontians). Benson [6] did suggest that this result could be due to the lack of postcranial information then available on eothyridids and basal caseids, a prediction borne out by the analysis of Reisz & Fröbisch [24] and our present analysis; the inclusion of *Eocasea martini* and the modifications presented here cause caseasaurs to once again be found as the sister to all other synapsids. Many of the characters which were put forward by Benson as synapomorphies of a clade containing Caseasauria, Edaphosauridae and Sphenacodontia are found to be absent in *Eocasea* and the new eothyridid material (Table 2).

**Table 2:** New information relevant to the relationships of Caseasauria included in the present analysis.

Character used by Benson to unite Caseasauria with Sphenacomorpha	New information from *Eocasea*, *Oedaleops* and *Vaughnictis*
Character 92, state 1 –presence of a prominent lateral process of supraoccipital	Absent in *Eocasea*
Character 131, state 1 –coronoid eminence formed laterally by the dentary	Formed laterally by the coronoid in *Eocasea* and *Vaughnictis*
Character 158, state 1 –dorsal transverse processes extend far laterally	Do not extend far laterally in *Eocasea* or *Vaughnictis*
Character 172, state 1 –three or more sacral vertebrae	Only two in *Eocasea*
Character 173, state 1 –first sacral rib of similar size to posterior sacral ribs	First sacral rib much broader in *Eocasea*
Character 191, state 1 –posterior margin of interclavicle head grades gradually into the shaft	Interclavicle head emarginated posterolaterally in *Oedaleops*
Character 215, state 0 –medial surface of ilium flat or weakly concave	Prominent ridge is present in *Eocasea*
Character 233, state 0 –anterior condyle of femur dorsoventrally compressed	Condyle is thick in *Eocasea*, *Odaleops* and *Vaughnictis*
Character 239, state 1 –Calcaneum length conspicuously greater than width	Length approximately equal to width in *Eocasea*

A summary of the characters previously supporting the grouping of Caseasauria with Sphenacomorpha by Benson [6].

This analysis supports the assertion by Spindler et al. [34] that *Datheosaurus* and *Callibrachion* are basal caseasaurs rather than haptodontine-grade synapsids. *Datheosaurus*, *Callibrachion* and *Eocasea* are found to be the successive outgroups to those caseids tested by Benson [6]. Spindler et al. did not test their hypothesis with cladistic analysis, but a number of the morphological features they identified support such an affinity in this analysis. For example, in *Datheosaurus* the large pineal foramen is located at the parietal mid-length, the robust mandible and dorsal ribs are curved throughout their length, and in *Callibrachion* the ectepicondylar foramen is present on the humerus and the dorsal process of the ilium is expanded anteriorly. The robusticity of the dorsal ribs in both was also commented on by Spindler et al. However one should note that Caseidae, as defined here, is a poorly supported clade. In fact, poor support is found throughout the tree except within Varanopidae and Edaphosauridae, and the CI score of the entire tree is low.

Lack of resolution within the strict consensus is found within Caseidae, Sphenacodontidae and Varanopidae. The iterative reduced positional congruence method [41], implemented in TNT, was used to identify the three wildcard taxa: *Caseopsis agilis*, *Ctenorhachis jacksoni* and *Angelosaurus dolani*. After pruning these taxa, 8 MPTs remained (Fig 10B). These three wildcard taxa were subjected to the analyses proposed by Pol & Escapa [41] to ascertain the reason for their lack of stability: inability to score potentially relevant characters due to the incompleteness of the fossils, or different characters suggesting conflicting relationships. The ancestral condition of unscored characters in the unstable taxa was examined in each MPT; if the optimisation of an unscored character varies between different MPTs, then the missing information in this character could have provided greater resolution. Meanwhile, scored characters may be examined by comparing the length of the character when the score for the unstable taxon is replaced by a missing entry. If the change in length differs between different MPTs, this character is supporting conflicting positions for that taxon. These analyses were carried out in TNT using the scripts provided by Pol & Escapa [41].

In all three of the wildcard taxa, numerous characters were identified that could contain potentially relevant missing entries: 11 for *A*.*dolani*, 52 for *Caseopsis* and 65 for *Ctenorhachis*. Character conflict was also found to influence the loss of resolution in two of these taxa: *Caseopsis* and *Ctenorhachis*. The character conflict in *Caseopsis* is between characters 171 and 233. The cup-like articular facet of the dorsal rib tuberculum of *Caseopsis* (character 171, state 2) is characteristic of the caseid clade containing the genera *Angelosaurus* and *Cotylorhynchus*. However, the anterior condyle of the femur of *Caseopsis* is not compressed (character 233, state 0), which supports a more basal position within Caseidae; all caseids more derived than *Casea* have a compressed anterior condyle. The dorsal vertebral morphology of *Ctenorhachis* supports conflicting positions. The ventral surface of the dorsal centra is strongly keeled, a characteristic of sphenacodontid synapsids (character 156, state 2). However, sphenacodontid synapsids and therapsids both have longer cervical centra than dorsal. In *Ctenorhachis* they are of similar length (character 153, state 1), a more plesiomorphic morphology found in haptodontine-grade sphenacodontians.

Due to the poorly supported relationships and lack of resolution provided by parsimony analysis, two alternative methods were used to assess the phylogenetic relationships among pelycosaurian-grade synapsids. The first was Bayesian analysis, the use of which is controversial in analyses of morphology [42], but some morphological systematists have suggested it should be preferred over parsimony [43,44]. One reason cited is that Bayesian analysis, which takes into account branch lengths, is less affected by issues such as long-branch attraction [45]; longer branches are more likely to convergently evolve characters and therefore cluster together under parsimony.

The second alternative method used was an implied weights analysis [46]. This method is a modification of maximum parsimony: all characters start equally weighted, but during each tree search characters which change state more than once are down-weighted each subsequent time a change occurs. Again, the use of this method has been controversial, mainly due to a sentiment that the weighting of characters is unparsimonious [47–49]. Nevertheless, it has been shown that using this weighting scheme produces better supported relationships [50].

The Bayesian analysis was carried out in Mr Bayes version 3.2.2 [51] using the Markov model [52] with a gamma distributed rate parameter. Searches were undertaken 10,000,000 times, with a quarter of trees discarded as burn-in. Two analyses were carried out: the first with the same matrix used in the parsimony analysis, and the second including 109 autapomorphous characters (S2 Appendix); since Bayesian analysis takes into account branch lengths, autapomorphies are necessary information [53]. For both analyses a majority rule consensus was constructed from the probability distribution of trees. The implied weights analysis was undertaken in TNT, using the same settings as in the parsimony analysis. Three analyses were carried out with different weighting schemes: homoplasious characters were downweighted with a concavity constant of 3, 5 and 10 respectively (a higher concavity constant is closer to equal weights).

**Fig 11:**
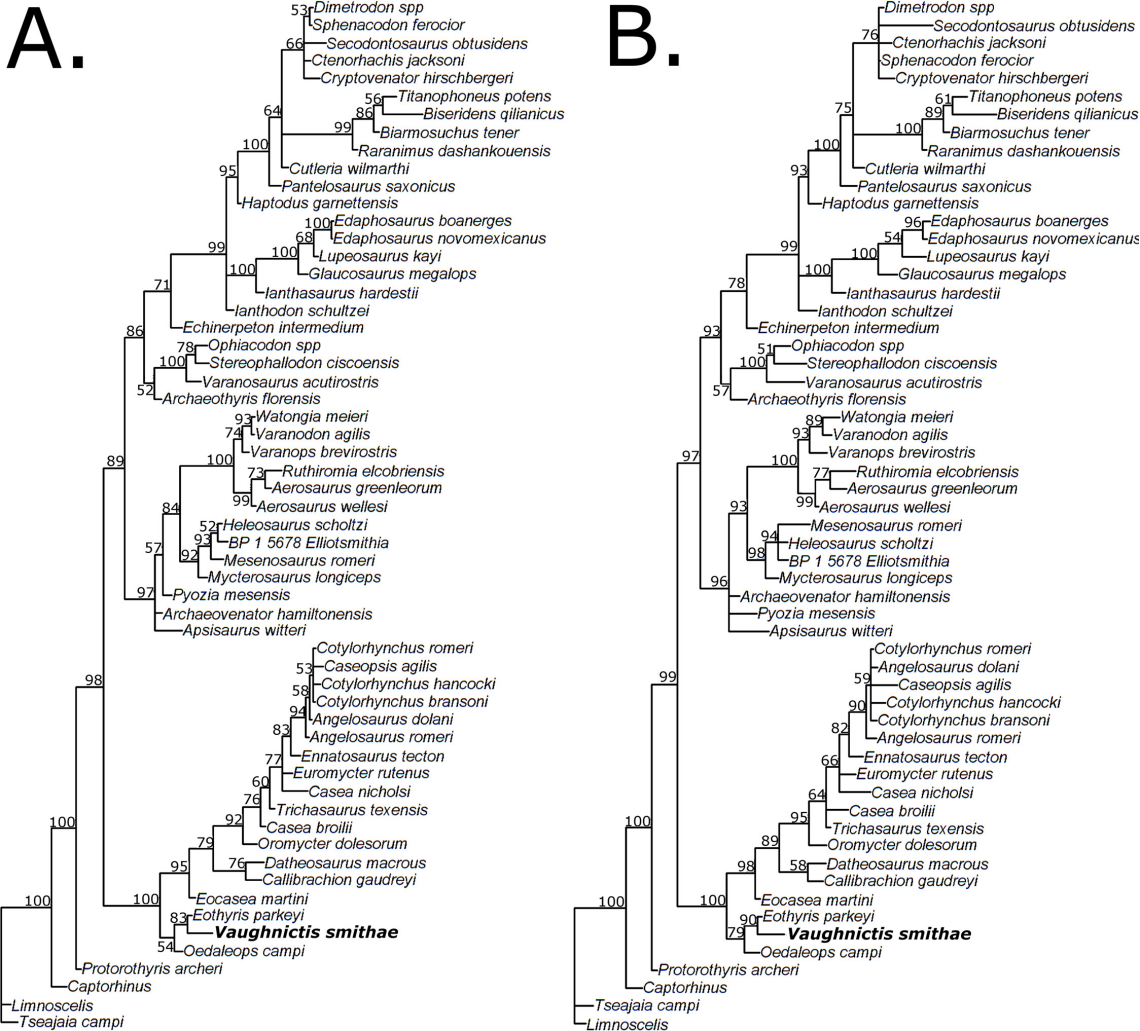
Results of phylogenetic analysis of the data matix using Bayesian analysis. (A) Analysis using the original matrix; (B) Analysis including autapomorphies. Branch lengths represent relative inferred character change. Numbers at nodes represent clade credibility values.

The implied weights and Bayesian analyses show broadly similar results to the parsimony analysis (Figs 10 and 11). Changing the concavity constant did not change the results of the implied weights analyses; the same 9 MPTs were found each time. An important discrepancy is in the position of *Echinerpeton*; both the implied weights and Bayesian analysis find this taxon to be the sister to the Sphenacomorpha. This change in position may reflect the fact that the characters uniting *Echinerpeton* with Ophiacodontidae are highly homoplasious. A relationship with the Sphenacomopha is suggested by the increased height of the neural spines and the flat medial surface of the ilium.

The support values for the phylogeny produced by implied weights analysis are for the most part higher than those found in the parsimony analysis, although the monophyly of Eothyrididae received lower support. The clade credibility values found in the Bayesian analyses indicate considerably higher support, with most being over 80% and many being over 90%. The positions of the wildcard taxa are better resolved in both the implied weights and Bayesian analyses; *Caseopsis* is resolved within the clade containing *Cotylorhynchus* and *Angelosaurus*, and *Ctenorhachis* is found in a basal position within Sphenacodontia. However, relationships of basal varanopids are not so well resolved in both Bayesian analyses.

The resolution of *Caseopsis* as a derived caseid within the *Cotylorhynchus/Angelosaurus* clade is particularly interesting given a recent study into caseid phylogeny [54]. This analysis included large numbers of continuous characters in an effort to resolve the positions of even the most incomplete fossils. In this study, *Caseopsis* was not found to be within the *Cotylorhynchus/Angelosaurus* clade, but was instead found in a more basal position. However, their data does provide an interesting perspective on the character conflict found within *Caseopsis*. The lack of compression of the anterior condyle of the femur is the plesiomorphic condition for caseids, and is absent in *Cotylorhynchus* and *Angelosaurus*. However, the lack of compression is also found in *Ruthenosaurus*, a derived casied found by the analysis of Romano & Nicosaia [54] within *Cotylorhynchus*. This would imply a reversal to the plesiomorphic condition, possibly demonstrating that this character is highly plastic. In fact, the compression of this condyle evolved independently in sphenacodontids and edaphosaurids. This may be the reason why *Caseopsis* is found to be more closely related to *Cotylorhynchus* and *Angelosaurus* in the implied weights analysis; this character, being highly homoplasious, is downweighted heavily. With this exception, the results of our analysis largely agree with those Romano & Nicosia [54], including the genus *Casea* being polyphyletic.

While these analyses and comparisons provide much to consider in future examinations of pelycosaur phylogeny, it is encouraging that the relationships obtained with the different methods are remarkably stable. Of particular interest in this paper, the relationships within caseasaurs and the eothyridid affinity of *Vaughnictis smithae* are consistent in all analyses. However, low resolution and support values within caseasaurs are also persistent. The poor support for a monophyletic Eothyrididae is particularly interesting considering the analysis of Sumida et al [7], who found this family to be paraphyletic. Considerably more work is needed before firm conclusions may be drawn about caseasaurian relationships. It has recently been shown that the fossil record of pelycosaurian-grade synapsids is limited and biased by sampling, gaps and incompleteness [55,56]. This is further supported by the above analyses, which regrettably show that the lack of resolution in the parsimony analyses is more due to incomplete specimens than conflicting characters. The issue of conflicting characters may be resolved by the addition of more characters or species that could provide valuable information on character polarities. There is unfortunately little that can be done to resolve the issue of incomplete data, at least until more specimens are found. However, the increased resolution provided by the implied weights and Bayesian analyses provide working hypotheses, which may be kept in mind during future examinations of these taxa.

## Supporting Information

S1 Appendix Changes made to the original data matrix of Benson. (DOCX)

S2 Appendix Autapomorphies used in the Bayesian analysis. (DOCX)

S1 Fig Close view of the left temporal fenestra. The damage by the original preparation is highlighted in a separate layer, allowing it to be toggled on and off.
(AI) Click here for additional data file.

S2 Fig USNM 22098. fe–Femur; dv–dorsal vertebrae.
(TIF)

S1 Matrix Character matrix used in phylogenetic analysis. (TRE)

## Abbreviations

MCZ: Museum of Comparitive Zoology, Harvard
USNM: Smithsonian National Museum of Natural History (formerly United States National Museum)
