# Supplementary material for: A Re-Description of ‘Mycterosaurus’ smithae, an Early Permian Eothyridid, and Its Impact on the Phylogeny of Pelycosaurian-Grade Synapsids
Source: PLoS One. 2016 Jun 22;11(6):e0156810. doi: 10.1371/journal.pone.0156810 (PMC4917111; doi:10.1371/journal.pone.0156810)
Supplement: S1 Appendix — (DOCX) [file pone.0156810.s001.docx]

**Supplementary Material 1**

Changes made to the character matrix of Benson (2012) for the phylogenetic analysis undertaken herein. With these exceptions, the character scores are unchanged from the nexus file included in the electronic supplement of the original paper.

Scores Changed

Character 25) *Eothyris* was originally coded as state 1 (dorsal process of premaxilla present). While there is a slight dorsal bulging of the maxilla, this is certainly not as extensive as the other taxa given the same character state. Therefore the score is changes to 0.

Character 29) The score of *Tseajaia* was changed to 1 due to it having only 11 maxillary teeth.

Character 34) *Oromycter* and *Oedaleops* were originally both coded as possessing a caniniform region (state 1). *Oedaleops* is hard to interpret due to the quality of preservation, but it appears that only two teeth were enlarged relative to the others, so its score was changes to state 2. *Oromycter* shows little evidence of heterogeneous dentition beyond a decrease in size in the posterior teeth, and so its score was changed to state 0.

Character 43) The presence or absence of denticles on the cutting edges of the teeth (ziphodonty) was updated based on the work of Brink & Reisz (2014) and Spindler (2015):

- *Dimetrodon* was coded as state 0; although denticles are present in *D. limbatus* and *D. grandis*, Benson (2012) stated that they primarily based the scores for *Dimetrodon* on *D. milleri* (this being the earliest and most plesiomorphic taxon), in which they are not present (Brink & Reisz 2014)
- *Sphenacodon* was changed to state 0 following Brink & Reisz (2014)
- *Archaeothyris* and *Echinerpeton* were changed to state 2 following Spindler (2015)

Character 59) The anterior process of the frontal was originally coded as long in both *Eothyris parkeyi* and *Oedaleops campi* (state 1) despite being shorter than the posterior process (Reisz et al., 2009). Both have been re-scored as state 0.

Character 119) *Mesenosaurus romeri* was originally coded as state 0 (posterolateral orientation of the transverse flange of the pterygoid), but the anterior orientation of the transverse flange was specifically noted by Reisz & Berman (2001). This taxon has been re-scored as state 1.

Character 140) *Ophiacodon* was originally scored as state 1 (contact present between splenial and posterior coronoid); this score was changed to 0 following observations by Spindler (2015).

Character 228) *Stereophallodon* *ciscoensis* and *Mycterosaurus longiceps* were both originally scored as character state 0 (prominent ventral ridge system on the femur). However, the poorly developed ventral ridge system has been considered characteristic of both these taxa (Brinkman & Eberth 1986, Berman & Reisz 1982). Therefore both have been re-scored as state 1 (ventral ridge system low and feebly developed)

Character modified

Character 17 – This character refers to the premaxillary tooth count. Benson (2012) divided it into two character states: state 0 representing 2-4 premaxillary teeth and state 1 representing 5-6 premaxillary teeth. However, these states miss some of the evolutionary variation. Several of the outgroup taxa, as well as some of the more basal members of the clades have four premaxillary teeth. It is possible that four premaxillary teeth is the primitive condition. By including the possession of four premaxiliary teeth in the character state representing fewer teeth, this character does not acknowledge reductions in tooth count e.g. in caseasaurs. Therefore the number of character states of character 17 have been increased to three: 0) 2-3 premaxillary teeth; 1) 4 premaxillary teeth; 2) 5-6 premaxillary teeth.

Characters Added

Character 240) Temporal fenestra morphology: 0) narrower dorsally than ventrally; 1) dorsal and ventral margins of similar length, fenestra has an oblong shape; 2) narrower ventrally than dorsally.

Character 241) Webbing under transverse processes of dorsal vertebrae: 0) webbing extensive, extends distally beyond the distal extent of the tips of the transverse processes and anteriorly to the forward edge of the vertebra; 1) webbing slight, does not extend distally beyond the distal extent of the tip of the transverse process; 2) webbing absent.

Character 242) Number of dentary teeth: 0) 23 or less: 1) at least 24 (Sidor 2003, Spindler 2015).

Character 243) Posterior extent of the anterior caudal ribs: 0) ribs long, extend posteriorly for the length of at least two caudal vertebrae; 1) ribs short.

Character 244) Dorsal ribs: 0) slender, proximal diameter of the shaft less than 1/ the centrum width; 1) robust

Scores for new and modified characters

|  | **17** | **240** | **241** | **242** | **243** | **244** |
| --- | --- | --- | --- | --- | --- | --- |
| ***Tseajaia campi*** | 1 | ? | 0 | 0 | ? | 0 |
| ***Limnoscelis*** | 0 | ? | 0 | 0 | 0 | 0 |
| ***Captorhinus*** | 0 | ? | 0 | 0 | 0 | 0 |
| ***Protorthyris archeri*** | 0 | ? | 1 | 0 | ? | ? |
| ***Dimetrodon spp.*** | 1 | 0 | 1 | 1 | 1 | 0 |
| ***Secodontosaurus obtusidens*** | 2 | 2 | ? | 1 | ? | 0 |
| ***Ctenorhachis jacksoni*** | ? | ? | 1 | ? | ? | 0 |
| ***Sphenacodon ferocior*** | 1 | 0 | 1 | 0&1 | 1 | 0 |
| ***Cryptovenator hirschbergeri*** | ? | ? | ? | ? | ? | ? |
| ***Titanophoneus potens*** | 0 | 0 | 1 | 0 | 1 | 0 |
| ***Biarmosuchus tener*** | 2 | 0 | ? | 0 | ? | 0 |
| ***Raranimus dashankouensis*** | 2 | 0 | ? | ? | ? | ? |
| ***Biseridens qilianicus*** | 2 | 0 | ? | 0 | ? | ? |
| ***Pantelosaurus saxonicus*** | 2 | 0 | ? | 0 | 0 | 0 |
| ***Cutleria wilmarthi*** | 1 | 2 | ? | 0 | ? | 0 |
| ***Ianthodon schultzei*** | 1 | 0 | ? | 1 | ? | 0 |
| ***Haptodus garnettensis*** | 2 | ? | 0 | 1 | 0 | 0 |
| ***Edaphosaurus boanerges*** | 2 | 0 | 2 | 0 | 0 | 1 |
| ***Edaphosaurus novomexicanus*** | ? | ? | 2 | 0 | 0 | 1 |
| ***Lupeosaurus kayi*** | ? | ? | 2 | ? | 0 | 1 |
| ***Ianthasaurus hardestii*** | ? | 0 | 1 | 1 | ? | 0 |
| ***Glaucosaurus megalops*** | 1 | ? | ? | ? | ? | ? |
| ***Echinerpeton intermedium*** | ? | ? | 0 | 1 | ? | ? |
| ***Archaeothyris florensis*** | ? | ? | 0 | 1 | ? | 0 |
| ***Varanosaurus acutirostris*** | 2 | 2 | 0 | 1 | 1 | 0 |
| ***Ophiacodon spp.*** | 2 | 2 | 0 | 1 | 1 | 0 |
| ***Stereophallodon ciscoensis*** | 2 | ? | 0 | ? | ? | ? |
| ***Oromycter dolesorum*** | 1 | ? | ? | ? | ? | ? |
| ***Ennatosaurus tecton*** | 1 | 0 | 2 | 0 | 1 | 1 |
| ***Eothyris parkeyi*** | 1 | 1 | ? | 0 | ? | ? |
| ***Oedaleops campi*** | 1 | 0 | ? | ? | ? | 0 |
| ***Casea broilii*** | 1 | 0 | 2 | 0 | 1 | 1 |
| ***Cotylorhynchus romeri*** | 1 | 0 | 2 | 0 | 1 | 1 |
| ***Angelosaurus romeri*** | 1 | ? | 2 | 0 | ? | 1 |
| ***Angelosaurus dolani*** | ? | ? | 2 | ? | ? | ? |
| ***Caseopsis agilis*** | ? | ? | ? | ? | ? | 1 |
| ***Trichasaurus texensis*** | ? | ? | 2 | ? | ? | 1 |
| ***Cotylorhynchus hancocki*** | ? | ? | 0 | 0 | 1 | 1 |
| ***Cotylorhynchus bransoni*** | ? | ? | 2 | ? | ? | ? |
| ***Euromycter rutenus*** | 1 | 0 | ? | ? | ? | ? |
| ***Wantongia meieri*** | ? | 0 | 1 | ? | ? | ? |
| ***Ruthiromia elcobriensis*** | ? | ? | 1 | ? | ? | ? |
| ***Varanodon agilis*** | 2 | 0 | 1 | ? | ? | ? |
| ***Varanops brevirostris*** | 2 | 0 | 1 | 1 | 0 | 0 |
| ***Aerosaurus wellesi*** | 1 | 0 | ? | 1 | 0 | 0 |
| ***Aerosaurus greenleorum*** | ? | ? | ? | ? | ? | ? |
| ***Archaeovenator hamiltonensis*** | 0 | 1 | 1 | 1 | 1 | 0 |
| ***Mesenosaurus romeri*** | 2 | 2 | ? | 1 | 1 | 0 |
| ***Mycterosaurus longicepos*** | 1 | 1 | 1 | ? | ? | ? |
| ***Heleosaurus scholtzi*** | 2 | 1 | 1 | ? | ? | 0 |
| ***BP-1-5678 Elliotsmithia*** | 0 | 1 | ? | ? | ? | ? |
| ***Pyozia mesenensis*** | 0 | ? | ? | ? | ? | ? |
| ***Eocasea martini*** | ? | ? | 2 | ? | 0 | 0 |
| ***“Casea nicholsi*** | ? | ? | 1 | ? | ? | 0 |
| ***Vaughnosaurus smithae*** | ? | 1 | ? | ? | ? | 1 |
| ***Apsisaurus witteri*** | ? | ? | 1 | ? | ? | 0 |
| ***Datheosaurus macrous*** | ? | ? | ? | ? | 1 | 1 |
| ***Callibrachion gaudreyi*** | ? | ? | ? | ? | ? | 1 |

Added Material

Six species have been added to the matrix of Benson (2012): *Casea nicholsi*, *Eocasea martini*, *“Mycterosaurus” smithae*, *Datheosaurus macrous*, *Callibrachion gaudryi* and *Apsisaurus witteri*. New material was also considered for *Oedaleops campi*. Here are presented the character scores for all seven of these species:

*Oedaleops campi*: ?20001100000010100??1000000?01000200??1010?0??01000000000001000000010??210?0?0000?011001?1110??????0????????????????????????0?1???????000?????????????????1???????00?0?000????0100???000?100010000??0300??????????1000?0?10??0?100?00?000??????0???01?0???00?0????00000????00?0?000000????0????0?00???0?0000?00?0??0?0?0000??0000???????0?0??0??0000???000???0000

*Eocasea martini*: ?????110?010??????????????0?????????????000?0??????????????????000?100??000000000001?201010000?12??00???????????????????????0????000????0?????0?0??1??200000?00000000000010000?????????????????????????????????0??????112?01?0?000??0??000?1??0?0?000??????????0??????????0?0???00000???00??????0?0??0?0????????0??????0???00???0????0000?000?000??0?0???0000?0??

*“Casea” nicholsi*: ???????????????????????????????????????????????????????????????0????0?????????0???010??????????????????????????????????????????????????????????????11????????11?0000000001?11???????????????????????????1001110011??1??1???????0000?0?0000????0?0??0????????????????????????????00???????????????????00??????????00???0????0??????????????100?00?????0????????0??

*Vaughnosaurus smithae*: ?????1000?00??????????????0??3?????????010????????1??00???0?0010000000320010?01????????1?????????????????????????????????????0??000??1000???0?????????????10?01?000????0?00????????????????????????????????????????????????????000100100001????1???1?????????????????0??????0?0??0???0??????????0?0???0??????????????0?000?????00???????0?0????????00?0?0?00?000?

*Apsisaurus witteri*: ?????1??0??1??????????????0??0?????????010?00?????????????????????????021?00100??1??????????????2??????001??0????0??00000????????000????0?1?????????????2112?0000?00?0000000?001001??0????000?00?0??000???????????????????11????001101000???????0??0?0?????????0?????0?0?0????00??????0?00???0??0??0000?????????0000???0000????0?000????0????0???0?0000?0?00?0111

*Datheosaurus macrous*: 10????????0?0??????????01?0????????1???000?00?????????????????????????????????????011????????????????????????????????????????11?0???????????????????1???0????????????????1????????????????20???0??????00????????0?0??????????0?100?????????1??0???110?????????????0???????????0???0?????????????00????0????????????0??0?000?0??0??????????0??????0??????0???00??0

*Callibrachion gaudreyi*: 1???????????????????1???1?0??????1?????000?0?????????????????????????????????????????????????????????????????????????????????2??0?????????1?????????????0????????????????01????0000??0?0??20001??000?10?????1000010????1????????00?0010????????????1??????????????0???????????0?????????????????00????0??????????0?0??0?000????0??????????0??0???0??0?0000??00??0
