# Supplementary material for: A Re-Description of ‘Mycterosaurus’ smithae, an Early Permian Eothyridid, and Its Impact on the Phylogeny of Pelycosaurian-Grade Synapsids
Source: PLoS One. 2016 Jun 22;11(6):e0156810. doi: 10.1371/journal.pone.0156810 (PMC4917111; doi:10.1371/journal.pone.0156810)
Supplement: S2 Appendix — (DOCX) [file pone.0156810.s002.docx]

**Appendix B**

Autapomorphies included in the Bayesian analysis of pelycosaurian-grade synapsids. Some of these autapomorphies represent specific characteristics within an already existing character. In these cases the relevant character was altered in the Bayesian analysis by adding an extra character state, and the autapomorphous taxon is the only taxon scored with this state. Such characters are noted below. In all other cases, an extra character was added to represent the autapomorphy, with the autapomorphous taxon coded as character state 1 and all others as character state 0, or “?” if the feature is not preserved.

*Oedaleops campi* – Parietal excludes postparietal from the posterior edge of the skull table

(Reisz et al. 2009)

*Eothyris parkeyi* – No precanine teeth in maxilla (Reisz et al. 2009) (requires modification of

character 32)

*Ennatosaurus tecton* – narrow parasphenoid body (Maddin et al. 2008)

– two large conical premaxillary teeth (Maddin et al. 2008)

– 5-7 apical serrations on marginal teeth arranged longitudinally

(Maddin et al. 2008) (requires modification of character 44)

*Ianthasaurus hardestiorum* – elongate cross-barred dorsal process on axis (Reisz & Berman

1986)

– at least 29 presacral vertebrae (requires modification of

character 148) (Reisz & Berman 1986)

– maximum of 8 lateral tubercles on each side of the neural spine

(Reisz & Berman 1986)

– proximal tubercles with ventral webbing (Reisz & Berman

1986)

– lateral tubercles lacking on posterior neural spines (or modify

character 169) (Reisz & Berman 1986)

*Glaucosaurus megalops* – prearticular extends to the jaw symphysis (Modesto 1994)

– septomaxilla exposed facially (also in *Haptodus*, *Varanodon* and

*Varanosaurus* and therapsids) (Modesto 1994)

*Edaphosaurus boanerges* – pterygoid tooth plate with 120-150 teeth (Modesto 1995)

– deeply excavated dorsal jaw symphysis (Modesto 1995)

– slender lateral tubercles (Modesto 1995)

*Lupeosaurus kayi* – transverse processes located high on the centrum (Sumida 1989)

*Edaphosaurus novomexicanus* – anisodont tooth plate (Modesto & Reisz 1992)

– reduced number of lateral tubercles on anterior presacral

neural spines (Modesto & Reisz 1992)

*Varanosaurus acutirostris* – step-like expansion of the ventral margin of the anterior maxilla

(Berman 1995)

– lateral and dorsal surface of snout separated by a sharp ridge

(Berman 1995)

– dorsal process of the premaxilla bifurcated (Berman 1995)

– septomaxilla exposed facially (also in *Haptodus*, *Varanodon* and

*Glaucosaurus*) (Berman 1995)

– Posterior process of jugal dorsoventrally narrow (also in

*Secodontosaurus*) (Berman 1995)

– middorsal margin of the quadratojugal forms the lateral wall of a

narrow, shallow, trough-like channel that is bounded medially by

the jugal and opens dorsally (Berman 1995)

– up to 56 maxillary teeth (requires modification of character 29)

(Berman 1995)

– basipterygoid fits into socket in the posteromedial flange of the

quadrate ramus of the pterygoid (Berman 1995)

– cultriform process long and narrow, reaches posterior boarder of

the internal naris (Berman 1995)

– cultriform process supported along its length by anterior ramus

of pterygoid (Berman 1995)

– dorsal process of stapes projects at right angle from the shaft

(Berman 1995)

*Haptodus garnettensis* – nasal overlaps tongue-like process of lacrimal (Currie 1977)

– notched supratemporal (Laurin 1993)

– robust, chisel-shaped teeth (requires modification of character 40)

(Laurin 1993)

– teeth on palatal ramus of the premaxilla (Laurin 1993)

– septomaxilla exposed facially (also in *Varanosaurus*, *Varanodon*

and *Glaucosaurus*)

*Secodontosaurus obtusidens* – skull roof elements thin (Reisz et al. 1992)

– parietal contributes to dorsal margin of the temporal fenestra

(Reisz et al. 1992)

– poorly developed supracanine buttress (requires modification

of character 37) (Reisz et al. 1992)

– Posterolateral wing of parietal reduced and directed posteriorly

(Reisz et al. 1992)

– Postparietal covers large area of supraoccipital (Reisz et al.

1992)

– reduction of dorsal and lateral processes of supraoccipital

(Reisz et al. 1992)

– reduction of lateral exposure of prefrontal (Reisz et al. 1992)

– posterior ramus of jugal dorsoventrally narrow (also in

*Varanosaurus*) (Reisz et al. 1992)

– transverse flange of pterygoid reduced in width (Reisz et al.

1992)

– Palatal exposure of palatine, vomer and ectopterygoid reduced

(Reisz et al. 1992)

– dorsal process of quadrate tilted anterodorsally (Reisz et al.

1992)

– lateral condyle of quadrate extends beyond lateral edge of skull

roof (Reisz et al. 1992)

– first dentary tooth directed forwards (Reisz et al. 1992)

– retroarticular process widely separated from reflected lamina

of angular (Reisz et al. 1992)

*Archaeothyris floresnsis* – ectepicondyle angled at 85 degrees to the plane of the distal

humerus (Reisz 1972)

– Well developed pubic tubercle (Reisz 1972)

*Pyozia mesenensis* – contact between quadratojugal and maxilla unexposed laterally

(Anderson & Reisz 2004)

– basipterygoid process anteriorly directed (Anderson & Reisz 2004)

– interpterygoid vacuity rounded anteriorly (Anderson & Reisz 2004)

*Heleosaurus scholtzi* – straight suture between premaxilla and nasal (Botha Brink & Modesto

2009)

– ornamented angular and surangular (Botha Brink & Modesto 2009)

*Varanops brevirostris* – maxilla dorsal process has anterior and posterior depression of

subequal size (Campione & Reisz 2010)

– postorbital with smooth transition from lateral to dorsal surfaces

(Campione & Reisz 2010)

– basipterygoid process hypertrophies (Campione & Reisz 2010)

– basipterygoid articular facets elongated mediollaterally (Capione &

Reisz 2010)

– posterior dorsal neural spines taper distally in lateral view (Campione

& Reisz (2010)

– presence of anterolateral excavation of femur proximal to fourth

trochanter (Campione & Reisz 2010)

*Archaeovenator hamiltonenesis* – medial process of postorbital underlying parietal (Reisz &

Dilkes 2003)

– broadly expanded nasal process of premaxilla (Reisz &

Dilkes 2003)

*Watongia meieri* – radius strongly curved (Reisz & Laurin 2004)

– enlarged pisiform (Reisz & Laurin 2004)

– reduced head of clavicle (Reisz & Laurin 2004)

*Mesenosaurus romeri* – premaxilla slender, forming a narrow rectangular snout in dorsal

view (Reisz & Berman 2001)

– long dorsal process of premaxilla forms anterior half of dorsal

margin of the external naris (Reisz & Berman 2001)

– lateral surface of premaxilla excavated, narrowing the base of the

dorsal process and expanding the narial shelf to extend nearly to the

snout tip (Reisz & Berman 2001)

– palatal process of premaxilla with elongated median suture (Reisz &

Berman 2001)

– well-developed depression in the lateral surface of the nasal

extending from narial border to the anterior end of the prefrontal

(Reisz & Berman 2001)

– slight lateral swelling of the maxilla above the canine (also in

– first premaxillary tooth shorter than second and third (Reisz &

Berman 2001)

– Vomerine teeth form a single median tooth row (Reisz & Berman

2001)

– Postorbital bar with nearly vertical posterior margin (Reisz &

Berman 2001)

– stapes rod-like with expanded quadrate process (requires

modification of character 94) (Reisz & Berman 2001)

*Varanodon agilis* – suspensorium far posterior, well back of occipital condyle (Olson 1965)

– well-developed antorbital fenestra (Olson 1965)

– fourth manual digit elongated and robust (Olson 1965)

– septomaxilla exposed facially (also in *Haptodus*, *Varanosaurus* and

*Glaucosaurus*)

*Ruthiromia elcobriensis* – posterior dorsal centra pinched laterally (hourglass shape in cross

section) (Brinkman & Eberth 1983)

*Aerosaurus greenleeorum* – proximal face of deltopectoral crest on the humerus is a large

triangular area (Romer 1937)

*Cotylorhynchus hancocki* – epicondyle of humerus broad, thin and platelike (Olson &

Beerbower 1953)

– attachment of M. pectoralis forms broad concave triangle that

flare to form a shelf-like ridge over surface of area of attachment

of M. coracobrachialis and M. brachialis (Olson & Beerbower

1953)

– first sacral rib greatly enlarged (Olson 1962)

*Cotylorhynchus bransoni* – astragulus with large foramen (Olson & Barghusen 1962)

*Caseiopsis agilis* – deepened acetabulum (Olson 1962)

– thickened pubis (Olson 1962)

*Angelosaurus dolani* – Femur with internal trochanter and ridge extending to proximal end of

intertrochantric fossa (Olson 1962)

*Mycterosaurus longiceps* – anterior ridge boardering intertrochateric fossa extends

proximally nearly to the end of the femur (Berman & Reisz

1982)

*Casea broilii* – supratemporal and tabular overhang squamosal to form a small but distinct

notch (Olson 1968)

– posterior tuber on the parasphenoid (Olson 1968)

– basipterygoid process forms two laterally projecting spurs (Olson 1968)

– basicranial articulation is mobile (Olson 1968)

– palatal teeth separated by deep grooves (Olson 1968)

– absence of stapedial foramen (Olson 1968)

– dorsal processs of stapes positioned more distally (Olson 1968)

– quadrate articulation of the side of the stapes (Olson 1968)

*Euromycter rutenus* – expanded temporal fenestra (Reisz et al. 2011)

– supernumerary blade-like intranarial bone located posteromedially to

the septomaxilla (Olson 1954)

*Casea nicholsi* – shafts of ribs expanded from the twelfth vertebra to the sacrum (Olson 1954)

*Angelosaurus romeri* – sacral ribs blade-like (Olson & Barghusen 1962)

– sacral ribs separated at articulation with ilium (Olson & Barghusen

1962)

*Lupeosaurus kayi* – strong posterior curvature of dorsal blade of scapula (Romer & Price

1940)

– ilium incised to receive sacral ribs (Romer & Price 1940)

– articular surface of sacral ribs concave (Romer & Price 1940)

– Puboischiadic plate broadened dorsoventrally (Romer & Price 1940)

*Ianthodon schultzei* – lingual fluting on marginal dentition (Kissel & Reisz 2004)

– anterior dorsal bulging of lacrimal (Kissel & Reisz 2004)

*Cutleria wilmarthi* – jugal-squamosal suture is anteriorly concave

*Pantelosaurus saxonicus* – Posterior end of the dentary well below posterior edge of the jaw

*Ctenorhachis jacksoni* – tapering tips of neural spines (requires modification of character

166) (Hook & Hotton 1991)

– posterior neural spines transversely compress posteriorly (Hook &

Hotton 1991)

*Dimetrodon* – Level of jaw articulation below dentary tooth row (Brink & Reisz 2014)

*Stereophallodon ciscoensis* – labial surface of maxilla extends down almost covering the

postcanine dentition (Romer & Price 1940)

– dorsal centra subrectangular in cross section (Brinkman &

Eberth 1986)

– lumbar vertebrae trefoil-shaped in cross section (Brinkman &

Eberth 1986)

– proximal fibula triangular (Brinkman & Eberth 1986)

*Ophiacodon* – Canines do not project laterally from the tooth row (Romer & Price 1940)

– Secondary adductor ridge on femur (Romer & Price 1940)

*Apsisaurus witteri* – anteroposteriorly elongate neural spines (Reisz et al. 2010)

– ovoid suborbital fenestra (Laurin 19991)

– interclavicle head broadened (Laurin 1991)

*Vaughnosaurus smithae* – posterior process of the maxilla extends beyond the posterior

margin of the temporal fenestra (observed)
