# Supplementary figures and images for: A Re-Description of ‘Mycterosaurus’ smithae, an Early Permian Eothyridid, and Its Impact on the Phylogeny of Pelycosaurian-Grade Synapsids
Source: PLoS One. 2016 Jun 22;11(6):e0156810. doi: 10.1371/journal.pone.0156810 (PMC4917111; doi:10.1371/journal.pone.0156810)

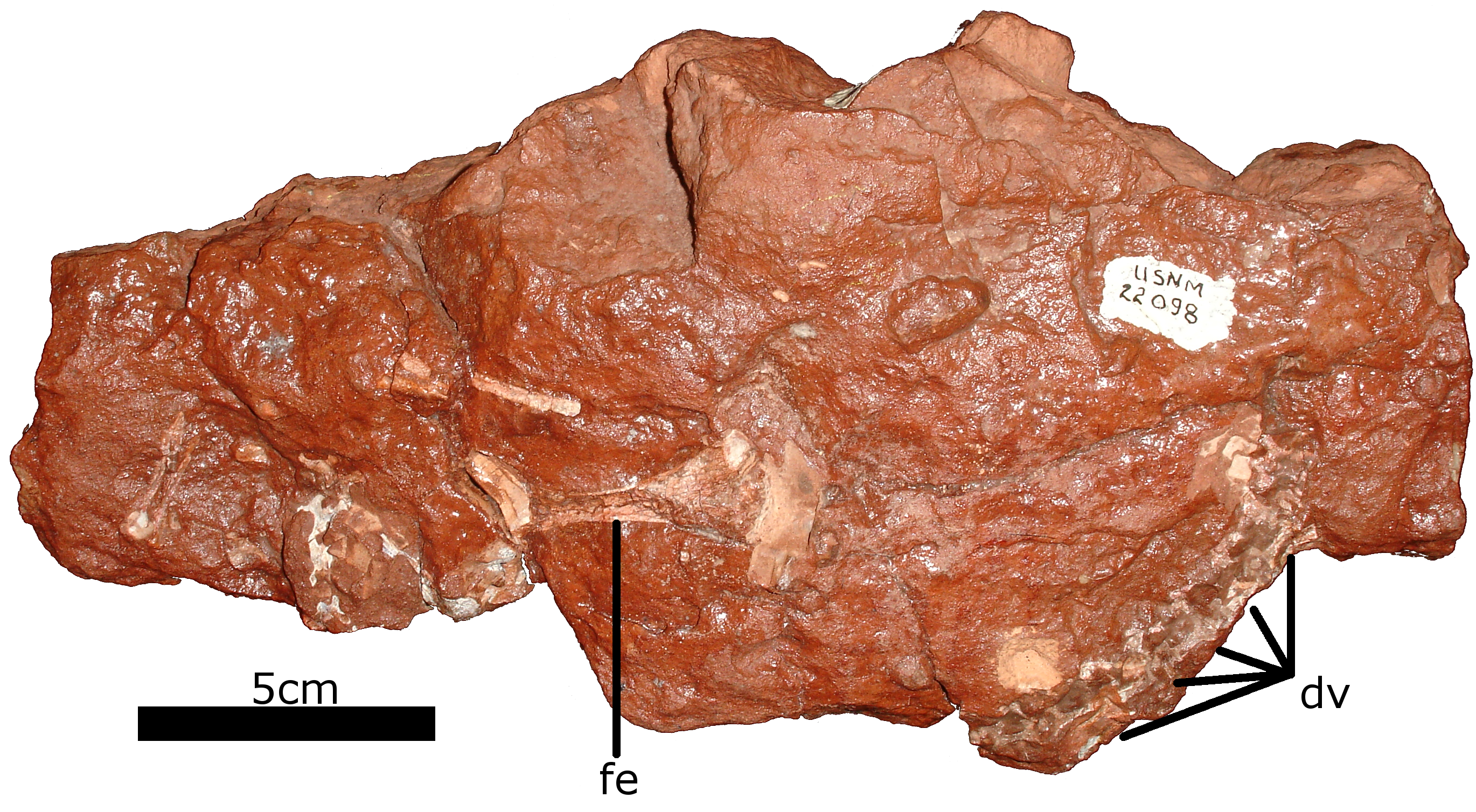

Supplement: S2 Fig — fe–Femur; dv–dorsal vertebrae. (TIF) [file pone.0156810.s004.tif]
